# The role of immune checkpoints in antitumor response: a potential antitumor immunotherapy

**DOI:** 10.3389/fimmu.2023.1298571

**Published:** 2023-12-15

**Authors:** Lidy Vannessa Mejía-Guarnizo, Paula Stefany Monroy-Camacho, Andrés David Turizo-Smith, Josefa Antonia Rodríguez-García

**Affiliations:** ^1^ Cancer Biology Research Group, Instituto Nacional de Cancerología, Bogotá, Colombia; ^2^ Sciences Faculty, Master in Microbiology, Universidad Nacional de Colombia, Bogotá, Colombia; ^3^ Medicine Faculty, Universidad Nacional de Colombia, Bogotá, Colombia

**Keywords:** immune checkpoint inhibitors, immune evasion, immunotherapy, HLA antigens, neoplasms

## Abstract

Immunotherapy aims to stimulate the immune system to inhibit tumor growth or prevent metastases. Tumor cells primarily employ altered expression of human leukocyte antigen (HLA) as a mechanism to avoid immune recognition and antitumor immune response. The antitumor immune response is primarily mediated by CD8+ cytotoxic T cells (CTLs) and natural killer (NK) cells, which plays a key role in the overall anti-tumor immune response. It is crucial to comprehend the molecular events occurring during the activation and subsequent regulation of these cell populations. The interaction between antigenic peptides presented on HLA-I molecules and the T-cell receptor (TCR) constitutes the initial signal required for T cell activation. Once activated, in physiologic circumstances, immune checkpoint expression by T cells suppress T cell effector functions when the antigen is removed, to ensures the maintenance of self-tolerance, immune homeostasis, and prevention of autoimmunity. However, in cancer, the overexpression of these molecules represents a common method through which tumor cells evade immune surveillance. Numerous therapeutic antibodies have been developed to inhibit immune checkpoints, demonstrating antitumor activity with fewer side effects compared to traditional chemotherapy. Nevertheless, it’s worth noting that many immune checkpoint expressions occur after T cell activation and consequently, altered HLA expression on tumor cells could diminish the clinical efficacy of these antibodies. This review provides an in-depth exploration of immune checkpoint molecules, their corresponding blocking antibodies, and their clinical applications.

## 1 Introduction

The amplitude and magnitude of immune responses are regulated by the interplay of co-stimulatory and inhibitory signals. Within this intricate orchestration, naïve CD4+T cells traverse the bloodstream and navigate through the spleen and lymphoid organs seeking professional antigen-presenting cells (APC) that, in the event of an infection, will present antigenic peptides on their HLA-II molecules. Self-peptides are presented on HLA-I molecules expressed on the surface of nucleated cells (1). In case of malignancy or intracellular infection foreign or mutated peptides will be presented on HLA-I molecules to CD8+ T cells.

**Figure 1:**
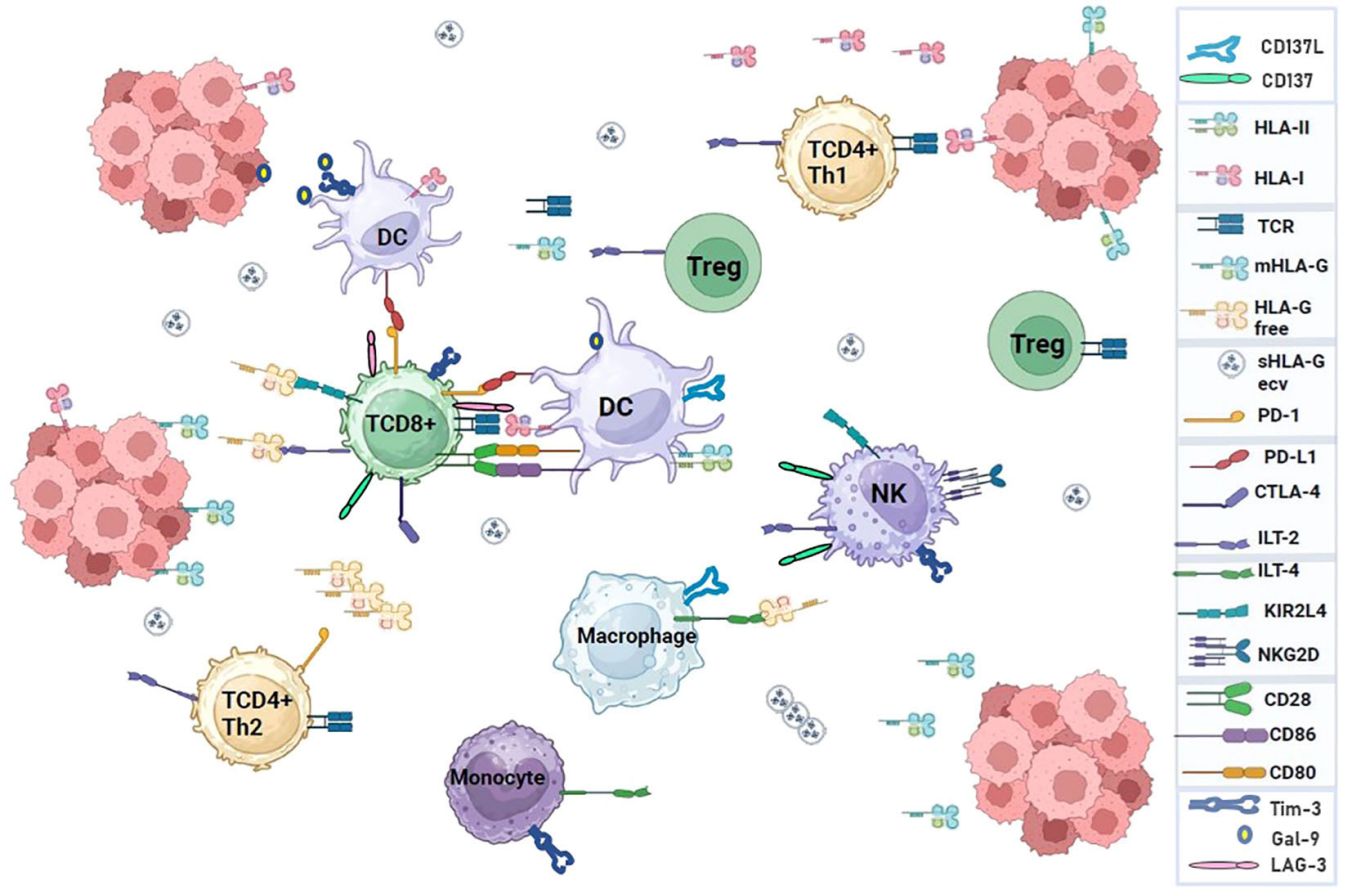
Interaction of immune system cell receptors and ligands in a tumor microenvironment. Immune checkpoint molecules can modulate the response of T cells to self-proteins, chronic infections, or tumor antigens. The pathways used by immune checkpoints are unique and non-redundant, demonstrating their important role in regulating immune homeostasis and highlighting the relevance of conducting research to develop immunotherapies based on multiple checkpoint blockades that enhance antitumor immunity.

The recognition of the HLA/antigenic peptide complex by the TCR is specific and provides the first signal of T cell activation. To continue its activation process, a second signal, given by the interaction between CD80 and CD86 molecules on the APC surface, with the CD28 molecule on the T cell surface (2, 3). Once activated, interleukin-2 (IL-2) and its receptor, IL-2R, were expressed by T cells to regulate its clonal expansion in an autocrine, or paracrine manner. This interaction stimulates T cell proliferation, increases survival and cell differentiation through cytokine production, increases energy metabolism, and overregulates survival genes. Once its effector function is exerted, immune checkpoint molecules are expressed to regulate T cell activation at different points during the immune response, maintaining the immune homeostasis (4, 5). This regulatory process, called peripheral tolerance, involves the activation of various immune checkpoint signaling pathways such as cytotoxic T cell-associated antigen 4 (CTLA-4), programmed cell death protein 1 (PD-1), Lymphocyte activation gene 3 (LAG-3), and T-cell immunoglobulin-3 (TIM-3), among others summarized in **Figure 1** and **Table 1**. Immune checkpoint molecules are essential for self-tolerance maintenance, protecting from an uncontrolled or continuous immune response that may cause tissue damage (5). The remarkable clinical efficacy of targeting immune checkpoints in various types of tumors has been demonstrated: CTLA-4 and PD-1/PDL-1 blockade has demonstrated significant success and are considered the most validated therapies related to immune checkpoints (6, 7). Given the relevance of this therapeutic approach, it is crucial to identify new immune checkpoint molecules on immune cells and develop antibodies to block them. Here, we highlight the expression of various immune checkpoint molecules, provide a summary of the main antibodies currently in development for targeting these immune check points. and review the action mechanisms of immune checkpoint proteins (**Supplementary Table 2**).

**Table 1:** Summary of the main characteristics of immune checkpoints.

Checkpoint	Inhibitory function	Structure	Cells of innate immunity on which it is expressed	Cells of adaptive immunity on which it is expressed	Ligands
PD-1	Regulates the activity of TL effectors in tissues and tumors. Essential in the balance of protective immunity, homeostasis, and tolerance.	A single IgV extracelular domain and contains 288 amino acids.	APCs Monocytes NK cells NKT cells Macrophages	Activated T cells and effectors, B cells	PD-L1 PD-L2
CTLA-4	Receptor homologous to CD28 in structure, with TL inhibitory function by competing with CD28 for binding to its ligands.	Contains a 36-amino acid cytoplasmic tail and a MYPPPY-type binding motif.	—--	FoxP3-induced Treg lymphocytes + or activated conventional T cells	CD80(B7-1) CD86(B7-2)
HLA-G	Important in immune tolerance in tissues. Tumor immune evasion mechanism.	HLA class I molecule no classic, typically composed of one α heavy chain, containing two peptide-binding domains (α1, α2), one immunoglobulin-like domain (α3), a transmembrane region and a cytoplasmic tail, as well as a light chain, a non-HLA encoded low molecular protein, named Beta-2 microglobulin (β2m). Each HLA-G subtype contains one to three spherical domains (α1, α2, and α3).	Various malignancies		ILT-2, ILT-4, and KIR2DL4
TIM-3	Inhibitory function. Induces tolerance in TLs, increases the secretion of suppressive cytokines (IL-10, TGF-β) by Treg lymphocytes, and enhances their suppressive activity. Increases cytotoxicity and IFN-γ secretion in effector NK cells and acts as a negative regulator of dendritic cell (DC) and macrophage function.	5 tyrosines in the cytoplasmic domain.	NK cells Macrophages DCs Mast cells Monocytes	Th1 and Tc1 cells Treg cells T cells B cells	Phosphatidyl-serine HMGB1 CEACAM1 (Galectin-9)
LAG-3	They control excessive activation after persistent antigen (Ag) exposure to prevent autoimmunity.	Type I transmembrane protein possesses four D1-D4 immunoglobulin (Ig)-like domains.	DCs NK cells NKT TAM	TILs CD4+ Tcells CD8+ T cells Treg cells	HLA - II Galectin-3 LSECtin
KIRs	They inhibit antigenic recognition and NK cell function.	Extracellular domains comprising two or three structural units of closely related immunoglobulin-like domains (D1 and D2 in most KIR2D receptors; D0, D1, and D2 in KIR3D receptors; and D0 and D2 in 2DL4 and 2DL5)	NK cells	TLs	HLA-I
(CD137) 4-1BB	TL-activating role; it promotes Th1 responses.	Glycosylated type I membrane protein composed of four cysteine-rich pseudo repeats (CRDs) forming the extracellular domain, a short helical transmembrane domain, and a cytoplasmic signaling domain	Activated NK cells, neutrophils, and mature DCs Monocytes Eosinophils	Stimulated and activated T-CD4+ and T-CD8+ cells, human B lymphocytes, and murine B lymphocytes	4-1BBL (CD137L/TNFSF9)

## 2 Expression and function of immune checkpoints in immune homeostasis

Adaptive immune response against cancer is mediated by CTLs that activate a mechanism of direct cytotoxicity resulting in tumor cell death. The strong immune pressure exerted by CTLs on tumor cells leads to the development of tumor variants with the capacity to escape immune recognition by deregulating the expression of classical and non-classical HLA-I molecules, which constitute the principal mechanism of evasion of the immune response used by the tumor (1). Antigen recognition leads to T cell activation and immune checkpoint expression, indispensable for maintaining homeostasis and self-tolerance (2, 3). These molecules are a category of receptors expressed on the surface of immune or tumor cells, which can either positively or negatively regulate immune responses (8). Within the tumor microenvironment, tumor cells exploit the overexpression of inhibitory receptors on immune cells that can result in T-cell exhaustion, characterized by diminished T-cell proliferation and reduced T-cell function, to avoid immune clearance (9).

Personalized therapy based on immune checkpoint blockade that can induce effective antitumor immunity, emerges as a challenge due to the large number of somatic mutations observed in most human tumors and the difficulty in finding new antigens with the potential to be recognized by the immune system (10). The first therapy of this type used in the clinic was the use of an anti-CTLA-4 antibody followed by others against PD-1 and PD-L1 which were approved by the US Food and Drug Administration (FDA) for clinical use. However, limitations, such as the development of adaptive resistance in most patients, unsatisfactory overall response rates, and adverse reactions like the development of autoimmunity, have difficult their clinic implementation (11). PD-1 (programmed cell death protein-1) CTLA-4 (cytotoxic T-lymphocyte-associated protein-4), TIM-3 (T cell immunoglobulin and mucin-domain containing-protein 3), LAG-3 (lymphocyte activation gene-3), HLA-G (Histocompatibility leukocyte antigen-G), Killer immunoglobulin-like receptors (KIR), and CD137 (4-1BB) are the main immune checkpoints under clinical development.

### 2.1 Cytotoxic T-lymphocyte-associated protein 4

**Figure 2:**
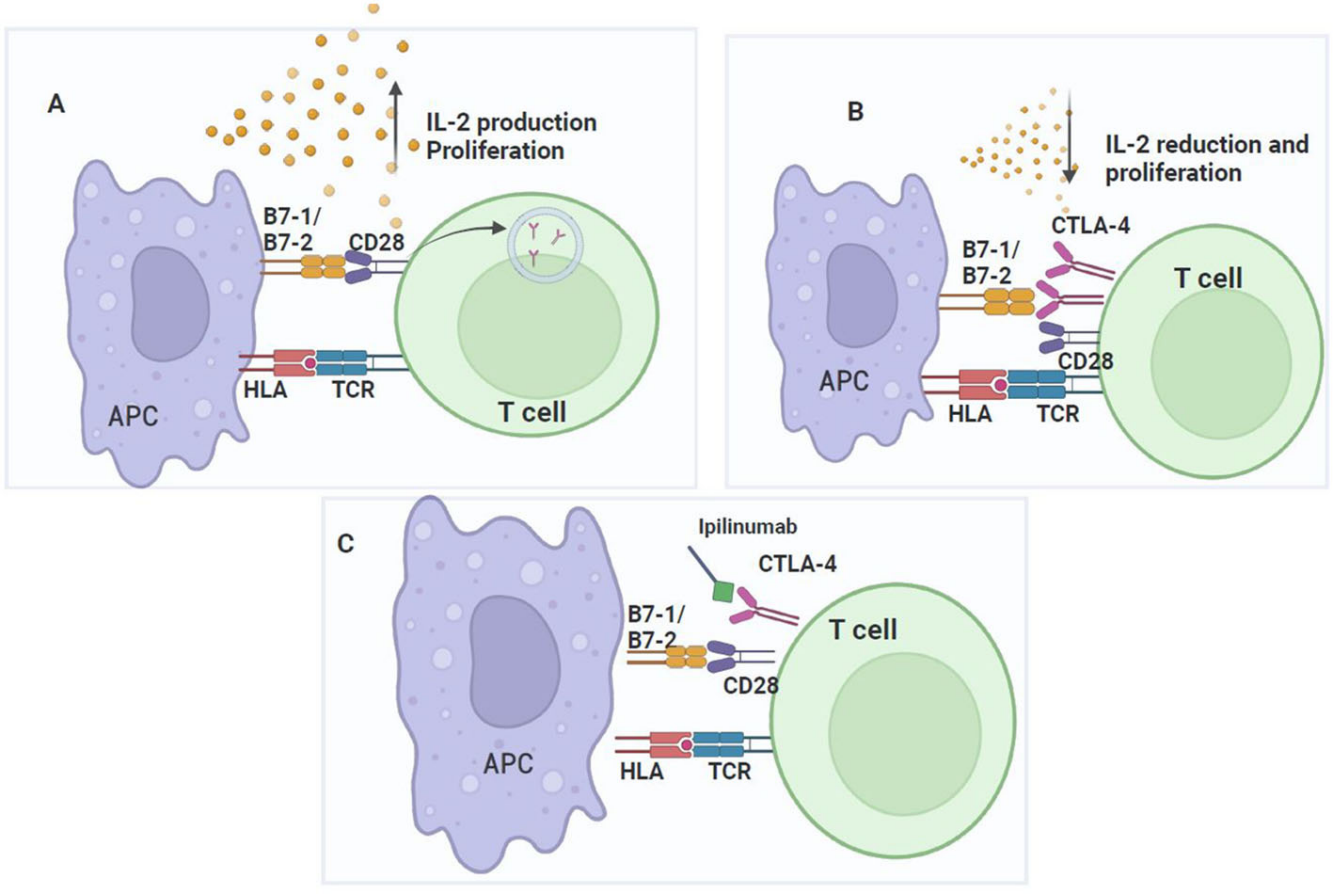
CTLA-4 checkpoint. CD28 signaling promotes T cell activation and CTLA-4 upregulation, which inhibits T cell proliferation. **(A)** T cell activation requires two signals: TCR binding to the HLA of the APC and co-stimulation given by the interaction of CD28 in lymphocytes with CD80/CD86 (B7-1/B7-2) in the APC, which increases IL-2 expression and the proliferation of antigen-specific T cells. During the activation process, conventional T-cells express low levels of CTLA-4, but upon complete activation, their expression increases on the cell surface. **(B)** CTLA-4 competes for binding to CD80/CD86 with a higher affinity than CD28. Once CTLA-4 binds to CD80/CD86, T cells activation is inhibited by disrupting CD28 signaling with CD80/CD86. Consequently, T cells proliferation is inhibited. **(C)** The mechanism of action of ipilimumab to block the binding of CTLA-4 to its ligands and prevent T cells inhibition.

CTLA-4 is an inhibitory transmembrane glycoprotein, homologous to CD28. These proteins, that belong to the immunoglobulin superfamily, are encoded by genes located on the long arm of chromosome 2 (2q) (12). CTLA-4 form homodimers, and as CD28, binds CD80 (B7-1) and CD86 (B7-2) (13), although with different affinities. After HLA/peptide recognition by the TCR, CD28 in the lymphocyte binds to CD80/86 in the antigen-presenting cell, providing the second signal for lymphocyte activation (**Figure 2A**). Co-stimulation by CD28 is essential for activating T cell responses, as without co-stimulation, an anergic, non-response, or cell death state is induced in the lymphocyte (14). On the other hand, CTLA-4, that binds to CD80/86 with higher affinity than CD28, has a dynamic role in the immune synapse by attenuating the early activating signals of T cells and inhibiting cytokine production and cell cycle progression to prevent autoimmunity (15). Although CD28 and CTLA-4 share the same ligands, they have opposite functions: CTLA-4 inhibits T cell activation while CD-28 activates it (5) and differ in expression, cellular localization, and lymphocyte trafficking (16, 17). In resting T cells, CD28 is constitutively expressed on the cell surface, while CTLA-4 levels are low, but when T cells activation occurs, CTLA-4 levels on the cell surface increase (5) (**Figure 2B**).

CTLA-4 inhibition of antitumor immunity is related to the CD28 expression. The cytoplasmic tail of CD28 contains signaling motifs, whose tyrosine residues, phosphorylated during T cell activation, bind to proteins containing SH2 domains, such as PI3K, GRB2, and SH3, such as the proline-rich protein PYAP. This interaction activates a downstream signaling pathways to activate transcription factors such as NF-κB and AP-1, involved in IL-2 production and T cell survival. On the other hand, CD28 co-stimulatory signal is indispensable for full TCR signaling and participates in cytoskeleton remodeling (18).

CTLA-4 is predominantly expressed in intracellular compartments such as lysosomes and endosomes of FoxP3+, regulatory T (Treg) cells or conventional activated T cells, so approximately 90% of CTLA-4 is intracellular (18). CTLA-4 molecules can either undergo recycling at the plasma membrane or degradation within lysosomal compartments (**Figures 2A, B**). Their cellular distribution depends on the phosphorylation status of their intracytoplasmic domains YVKM, with the transcription factor AP-1, which regulates CTLA-4 transport from the Golgi apparatus to the cell surface (16, 19). CTLA-4 transport to endosomes and lysosomes for storage or degradation is mediated by the interaction of cell surface CTLA-4 with AP-2. When the tyrosine residues in the intracytoplasmic domain of CTLA-4 are not phosphorylated, CTLA-4 undergoes degradation. Subsequently, upon TCR activation, CTLA-4 stored in lysosomes is released, leading to an increase in its expression on the T cell surface (15, 20).

CTLA-4 uses intrinsic mechanisms to affect CTLA-4 expressing T cells and extrinsic mechanisms to influence secondary cells in inhibiting T cell activation. Notably, when in competition with CD28, CTLA-4 primarily exerts its extrinsic effects. CTLA-4 is phosphorylated by the Src family of protein tyrosine kinases (PTK), after binding to CD80/CD86; then, it binds to phosphatidylinositol 3-kinase (PI3K) and activates SHP2 phosphatases, which, upon association with CTLA-4, alters the phosphorylation of the CD3-Z chain and limits TCR signaling. Recruitment of serine/threonine phosphatase 2A (PP2A) by CTLA-4 decreases downstream AKT phosphorylation and the T cell activation signal initiated by TCR binding to the antigen (16), counteracting the TCR and CD28-induced kinase signal (21). CTLA-4 inhibitory function can occur by independent (extrinsic) signaling mechanisms in a trans-endocytosis process, which removes surface CD80 and CD86 from the APC, to directly reduce the stimulating capacity of APCs through CD28. CTLA-4 exerts a pivotal role in immunity, as has been demonstrated, since CTLA-4 inhibition (**Figure 2C**) increases the CD4+ T cell-dependent immune responses and inhibits the immunosuppressive function of Treg cells, which constitutively express this immune checkpoint protein (21). Moreover, CTLA-4 knockout mice died at three to four weeks of life due to severe pancreatitis, myocarditis, and T cell infiltration into liver, heart, lung, and pancreas (21).

#### 2.1.1 Anti-CTLA-4 antibodies

Given that CTLA-4 blockade can lead to tumor regression in murine models (22, 23), multiple studies concluded with the clinical development and approval of anti-CTLA-4 monoclonal antibodies (mAb) for the treatment of patients with advanced melanoma (24, 25). The first drug to be developed against CTLA-4 was ipilimumab, a mAb capable of antagonizing CTLA-4 (**Figure 2C**), for its use alone or in combination, in the treatment of melanoma, prostate, breast, renal and other cancers (26). As phase III studies demonstrated the benefit of ipilimumab on the overall survival of patients with advanced melanoma, its use for melanoma treatment was approved by the FDA and the European Medicine Agency in 2010 (27). Subsequently, as clinical trial results demonstrated that approximately 20% of patients had an increased survival after ipilimumab therapy (28), its application in other types of cancer and its use in combination with other immune checkpoint inhibitor antibodies or treatment modalities, such as chemotherapy, radiation therapy, or immunotherapy, are currently under evaluation (29).

The second anti-CTLA-4 antibody being evaluated in clinical trials is tremelimumab, a humanized IgG2 mAb that binds CTLA-4 and blocks the interaction with its ligands, CD80 and CD86, to improve antitumor immune response mediated by T cells. Clinical trials using anti-CTLA-4 as monotherapy or in combination with durvalumab (a mAb against PD-L1) are underway in several countries in various types of cancer such as non-small cell lung cancer (NSCLC), head, neck, gastric, and pancreatic cancer, hepatocellular carcinoma, and several hematological cancers (30, 31). In October 2022, tremelimumab, in combination with durvalumab, was approved for the treatment of adult patients with unresectable hepatocellular carcinoma in the US (32). Tremelimumab has also been tested as a potential treatment for malignant mesothelioma and advanced melanoma (33).

### 2.2 Programmed cell death protein 1/programmed cell death protein 1-ligand 1 or 2

PD-1 is a 50-55 kDa type I transmembrane glycoprotein acting as an immune receptor of the CD28/CTLA-4 receptor family. It shares 15% sequence homology with CD28, 20% with CTLA-4, and 13% with ICOS (34), and unlike other CD28 family members, PD-1 is monomeric in solution and on the cell surface because it lacks the membrane-proximal cysteine residue required for homodimerization (35). PD-1 regulates effector T cell activity in tissues and tumors, unlike CTLA-4, which regulates the activation of naïve T cells in lymphoid organs. PD-1 is expressed on effector T cells in peripheral tissues or in the tumor microenvironment and other immune cell subpopulations, such as B lymphocytes, APCs, and NK cells.

PD-1 interaction with its ligands PD-L1 (B7H1 or CD274) and PD-L2 (B7DC or CD273) negatively regulates TCR signaling in T cells. These ligands are type 1 transmembrane proteins. PD-L1 is expressed constitutively on tumor and immune cells, or its expression can be induced by interferon γ (IFN-γ) and other cytokines production, in the tumor microenvironment, by activated T cells. Its biological significance includes aspects related to humoral immunity, immunity against infections, transplantation immunity, hypersensitivity, and immune privilege (36). PD-1 signaling begins when the HLA/peptide complexes on APCs surface are recognized by antigen-specific T cells that proliferate when become activated and exert their effector action.

**Figure 3:**
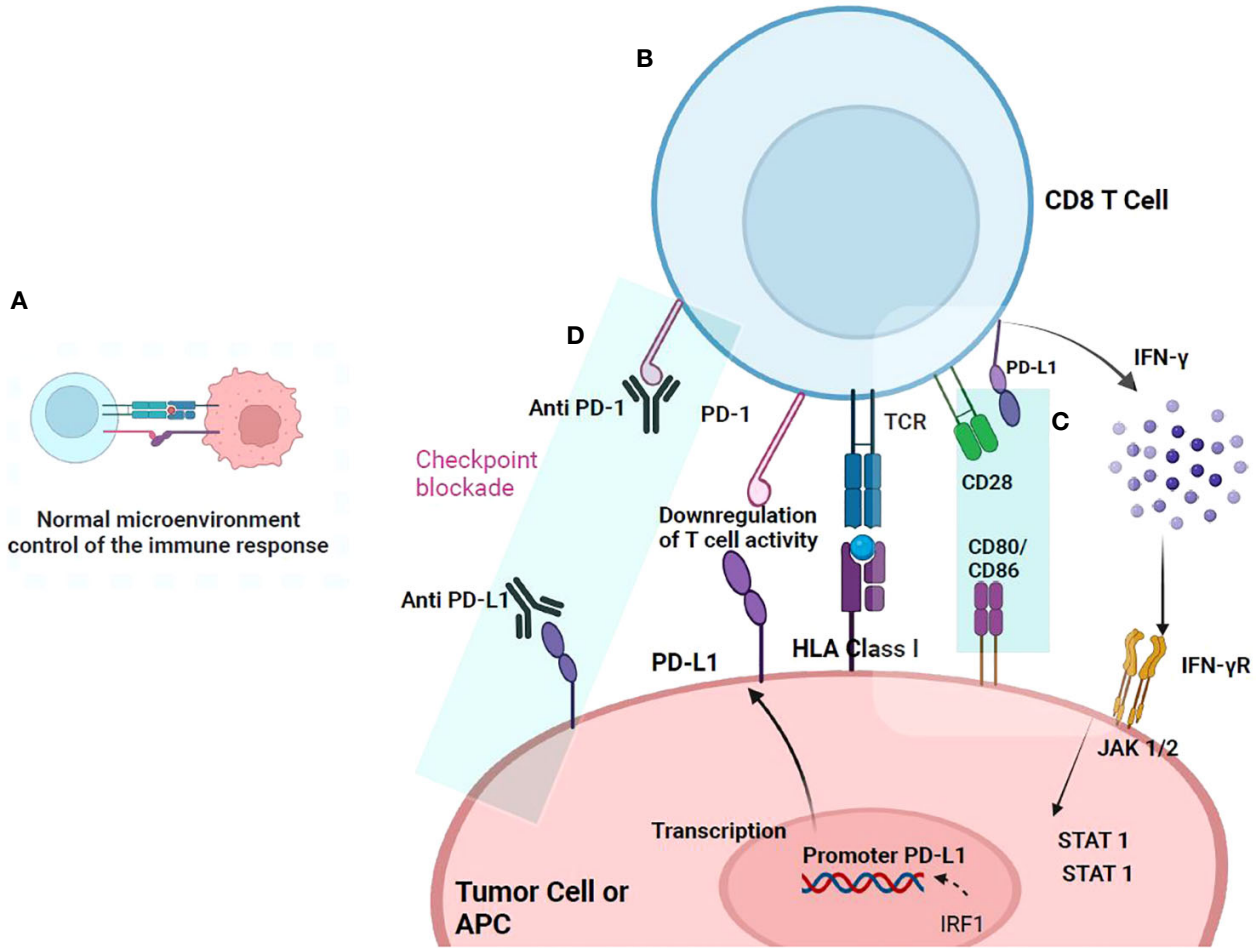
PD-1/PD-L1 checkpoint. The action mechanism begins when T cells are activated upon recognition of the antigen presented in HLA-I, triggering a signaling cascade and the release of cytokines that activate their proliferation. **(A)** In a normal microenvironment, after T cell activation and proliferation, immune checkpoint proteins, such as PD-1 and its ligand PDL-1, are expressed that prevent their excessive activation. **(B)** In a tumor microenvironment, the expression of immune checkpoints favors escape from immunosurveillance. Once T cells recognize the tumor antigen presented in HLA-I, they are activated and produce IFN-γ that binds to the IFN-γ receptor, inducing PD-L1 overexpression in tumor cells. PD-L1 binds to PD-1, which is overregulated in T cells, and thus inhibits the immune response. **(C)** PD-L1 can interact with CD80/CD86 on the antigen-presenting cell (APC) and disrupt PD-L1/PD-1 binding. It has been documented that CD80/CD86 are not only expressed on APCs but also on T cells, suggesting that it may be another pathway in T cells that may serve to downregulate responses and prevent T cell signals. **(D)** Anti-PD-1 antibody or anti-PD-L1 blocks the interaction of PD-1 and PD-L1 and suppresses the inhibition of CD8+ T cells, thereby enhancing antitumor activity.

In a normal microenvironment (**Figure 3A**), after T cell activation and proliferation, expression of immune checkpoints such as the PD-1/PD-L1 axis prevent excessive T cell activation against antigens and avoid autoimmunity (37). However, in the tumor microenvironment, immune checkpoints expression favors immune escape. In the tumor, after antigenic recognition, antigen-specific T cells release IFN-γ and activates T cell proliferation to increase tumor immune destruction (**Figure 3B**). IFN-γ release increases CD8+ T cell proliferation but overregulates PD-L1 expression on tumor and stromal cells. On the other hand, TCR signaling overregulates PD-1 expression on the T cell surface (38) (**Figure 3C**). PD-1 interaction with CD80 inhibits the T cell proliferation, because CD80 acts as an inhibitory signal when binds PD1 (**Figure 3C**). PD-1 interaction with any of its ligands leads to dephosphorylation and inactivation of the T-cell kinase ZAP70 and SHP2 recruitment. SHP2 dephosphorylates PI3K, inhibiting AKT activation (39), reducing the production of inflammatory cytokines, and of cell survival proteins such as Bcl-xL. CTLA-4 is highly expressed on Treg cells and, after binding to their ligand increases its proliferation and suppressive activity.

These findings demonstrate a unique and complex mechanism of action of PD-1/PD-L1, as knockout mice developed autoimmunity with elevated levels of IgG2b and IgA production, developed a mild lupus-like disease and a late-onset cardiomyopathy. However, these disease phenotypes were mouse strain-dependent and occurred late in life. Additionally, the autoimmune effects of PD-1 knockout mice were more severe than those observed in CTLA-4 knockout animals (40).

Anti-PD-1 therapy is a new way to inhibit immune checkpoints and has great potential for treating patients with refractory or recurrent tumors (**Figure 3D**). Research on human PD-1 has evolved since its discovery more than 20 years ago. First, the gene structure and genomic organization of PD-1 were investigated. Subsequently, the mechanisms that regulate the expression and immune checkpoint activity of PD-1 in coordination with its PD-L1 and PD-L2 ligands were studied (41).

The study of human PD-1 gene has been a success and has marked the course of current biomedical research, translating it from the laboratory to the clinic. However, the molecular mechanisms of regulation mediated by this gene are still undetermined. Clinical research has revealed a wide variability in response rates to PD-1 blocking therapy in different cancers, with percentages ranging from 18 to 87%. In addition, patients may acquire resistance to immune checkpoint inhibition therapy, and some ones may experience hyper-progressive disease after receiving anti-PD-1 therapy, A biomarker to predict individual patient response to this therapy has not yet been identified. In summary, although this immune checkpoint inhibition therapy has revolutionized the field of cancer immunotherapy, further efforts are still required to study the molecular mechanisms of immune checkpoints and to develop clinical research that allows personalized and precision medicine, to optimize the results of this therapy and improve the ability to predict patient response to immune checkpoint blocking immunotherapies (41).

#### 2.2.1 Inhibitory antibodies against PD-1/PD-L1

The success of ipilimumab has spurred the development of new antibodies against other immune checkpoints, such as those directed against the PD-1/PD-L1 axis. Antibodies like nivolumab, pembrolizumab, and pidilizumab are designed against PD-1, while atezolizumab and durvalumab target PD-L1. A promising avenue involves a clinical development focus on a fusion protein (PD-L2) designed to PD-1+ T cells (**Figure 3D**). Clinical trials focusing on the PD-1/PD-L1 axis have demonstrated superior response rates compared to CTLA-4 blockade (42), attributed in part to the milder adverse events associated with PD-1/PD-L1 blockade. Nevertheless, an evaluation of the antibody nivolumab (BMS-936558), revealed 14% of participants experiencing grade 3 or 4 immune-related adverse events, including three deaths due to pulmonary toxicity post-administration (43). In comparison with standard chemotherapy with dacarbazine or paclitaxel combined with carboplatin in melanoma patients unresponsive to ipilimumab, nivolumab exhibited a higher objective response rate (32% vs. 11%, respectively). Additionally, fewer toxic effects were observed in nivolumab-treated patients than those reported with existing chemotherapy regimens for advanced melanoma treatment (44). After ipilimumab, or its combination with a BRAF inhibitor, nivolumab emerged as a new treatment option with durable and clinically meaningful objective responses. This led to its FDA approval as a second-line treatment for liver cancer in 2017 (45).

Several therapeutic combinations have gradually been approved for the treatment of various types of cancer. In 2022, the FDA approved in the US the combination treatment of nivolumab with relatlimab (anti-LAG-3) for the treatment of unresectable or metastatic melanoma in adult and pediatric patients older than 12 years (46). Nivolumab combined with conventional chemotherapy or ipilimumab had a clinically significant increase in antitumor activity in patients with advanced gastroesophageal cancer, with a manageable safety profile (47). Due to its significantly higher antitumor activity, pembrolizumab was approved by the FDA in 2014 for the treatment of melanoma patients previously treated with ipilimumab (45). It has been shown to confer a favorable response as single therapy in hematologic cancers; its use in combination with other therapies, such as rituximab (anti-CD20) for the treatment of refractory NSCLC induced an overall response rate of 19 to 25% (48). On the other hand, in phase 2 and 3 clinical trials, an increase of 4.2 and 2.9 months in overall survival was observed, respectively, with the administration of atezolizumab (anti-PD-L1) compared to chemotherapy with docetaxel in patients with NSCLC (49).

### 2.3 Histocompatibility leukocyte antigen-G

**Figure 4:**
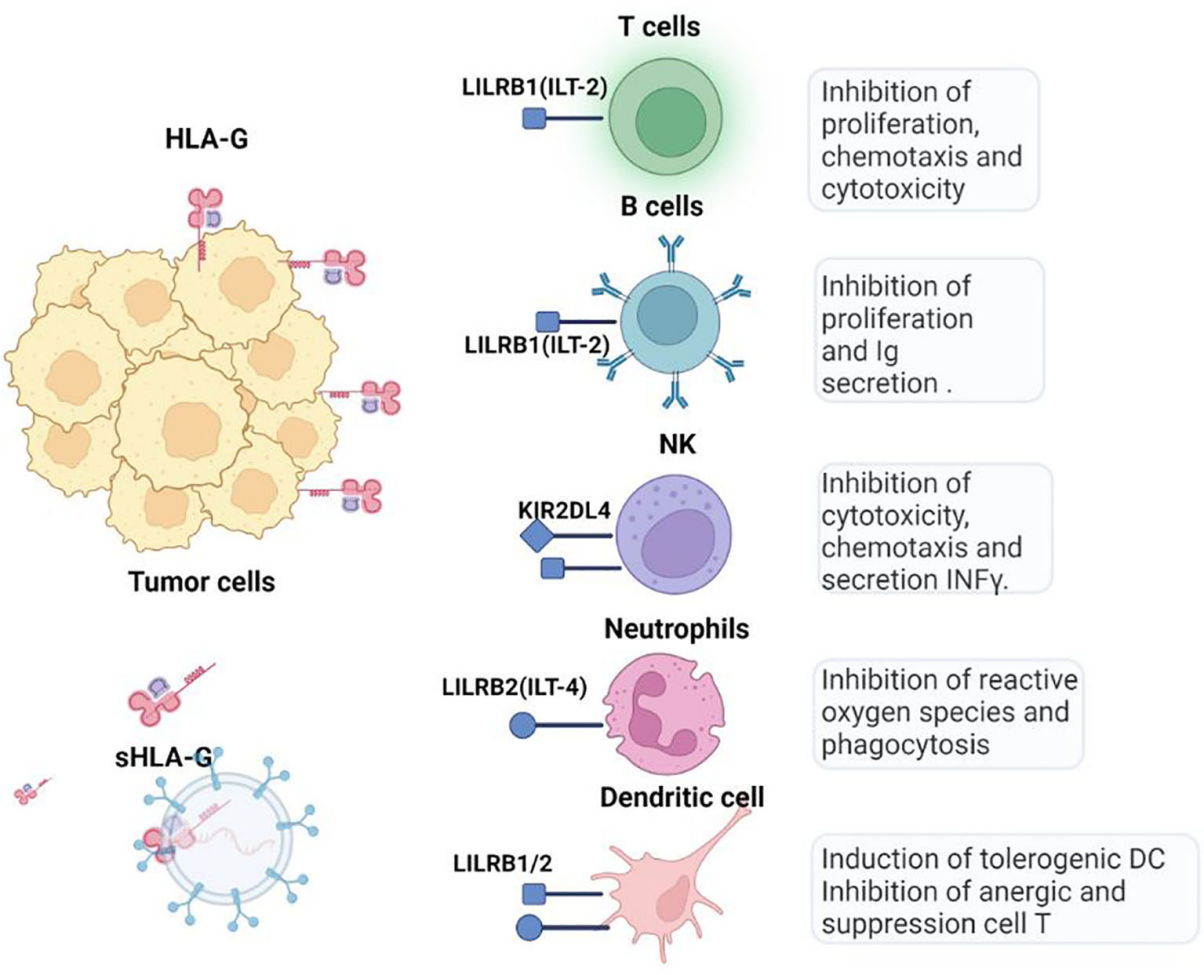
HLA-G checkpoint. Through the interaction of LILRB1 (ILT-2) and LILRB2 (ILT-4) receptors, HLA-G inhibits cytotoxic T cells, NK cells, and B cells and modulates myeloid cells. Soluble isoforms can be generated by proteolytic cleavage of membrane-bound HLA-G forms and move to other tissues via blood or extracellular vesicles.

Human histocompatibility leukocyte antigen (HLA-G) is a non-classical HLA-I molecule located at chromosome 6p21.3 within the region encoding the major histocompatibility complex (MHC). Although it has the same structure as classical HLA class I molecules, its main function is not antigen presentation (50) HLA-G modulates immune system functions in immune privileged tissues such as the maternal-fetal interface, thymus, and cornea. HLA-G expression during pregnancy avoids the fetus immune destruction by the mother’s immune system (51), playing a crucial role in maternal-fetal tolerance. Under normal physiological conditions, HLA-G is not expressed in adult tissues; however, aberrant expression of HLA-G anchored on the cell surface, released into plasma as a soluble form, or as part of exosomes has been observed in most human tumors analyzed but not in surrounding healthy tissue (52). HLA-G expression has been observed in most human tumors analyzed to date, but not in surrounding healthy tissue (53). However, the degree of HLA-G expression varies between tumors or within the same tumor, possibly due to the polymorphism exhibited by this molecule. Seven HLA-G isoforms has been documented for HLA-G: four membrane associated (HLA-G1, G2, G3, and G4) and three soluble (sHLA-G5, G6, and G7), generated by alternative splicing (54), and as all HLA-G molecules expressed on the cell membrane can be detached, by the action of metalloproteases or can be secreted, this structural diversity is even greater (52) (**Figure 4**). The heterogeneous pattern of HLA-G expression in tumors is probably the main obstacle in the clinical response to immunotherapy with immune checkpoint inhibitors. Although in colorectal and esophageal cancer, a high variability in the expression levels of HLA-G and its receptors in different areas within the tumor has been demonstrated, it has not been determined which of these isoforms plays a functional role in cancer immunology, nor how the different isoforms relate to ILT-2, ILT-4, and KIR2DL4 receptors in immune cells, or what are the effects of these interactions. This variability has also been observed when using different anti-HLA-G antibodies and thus, based on the above and considering that HLA-G is an important immune checkpoint, HLA-G expression and its clinical significance must consider intratumor heterogeneity, and the different specificities of the anti-HLA-G antibodies used (55).

HLA-G exerts inhibitory effects on innate and adaptive antitumor immune cells by binding to its ILT-2 (expressed on monocytes, B cells, dendritic cells (DCs), a subset of NK cells and T cells), ILT-4 (specific to myeloid cells), and KIR2DL4 receptors, (expressed on NK cells) (56) (**Figure 4**). HLA-G inhibits the cytotoxic function of NK cells in uterine and peripheral blood, as well as the cytotoxic functions of CTLs and γδ T cells Additionally, it dampens the alloproliferative response of CD4+ T cells and inhibits the proliferation of T cells and peripheral blood NK cells. Moreover, HLA-G hinders the maturation and functionality of DCs and induces the differentiation of T cells into Treg and myeloid suppressor derived cells (MDSCs). Unlike CTLA-4 and PD-1, HLA-G interferes at all stages of the antitumor immune response, from impairing the APCs activation, to hindering and the presentation and activation of effector cells affecting the function of activated CTLs and NK cells.

In cancer, there is a correlation between HLA-G expression in both, tumor and tumor-infiltrating cells and unfavorable prognostic factors including higher tumor grade (57). Studies in animal models have shown that the use of anti-HLA-G blocking antibodies can effectively restore immunity against HLA-G-expressing tumor cells *in vivo*. Furthermore, HLA-G expression is associated with metastasis and poorer survival in a humanized murine model of ovarian cancer (58), playing a key role in fostering immune tolerance and constitutes an escape mechanism of tumor cells (59). In preclinical immunocompetent models, the HLA-G expression in human ovarian carcinoma cell lines has demonstrated an increased capacity for migration and invasion compared to their HLA-G negative counterpart in nude mouse models. HLA-G-positive cells exhibited a tendency towards widespread metastasis leading to diminished survival rates in xenograft models, demonstrating the role of HLA-G as an immune checkpoint molecule that may influence tumor cell invasion and metastasis (56). HLA-G seems to be a promising target for immunotherapy. Although the relevance of HLA-G in cancer incidence and development has been demonstrated, characterizing the pattern of this neo expression remains challenging due to the absence of antibodies against the less studied isoforms (HLA-G2, G3, G4, G6, and G7) (54). It is worth noting that only 4H84, MEM-G1, and MEM-G2 antibodies can recognize all HLA-G isoforms, but it is important to consider that 4H84 and MEM-G1 exhibit cross-reactivity with proteins other than HLA-G and bind to antigen-free classical HLA-I heavy chains on activated lymphocytes (60). Consequently, to determine the expression levels of different HLA-G isoforms remains elusive based on the use of specific antibodies (61).

#### 2.3.1 Inhibitory antibodies against HLA-G

Although HLA-G may be an important target for immune checkpoint inhibition therapy, mAbs directed against HLA-G are few. Their non-therapeutic clinical use has not been developed because it is unclear whether these antibodies can effectively block HLA-G binding to ILT-2, ILT-4, and KIR2DL4, given that most of them recognize only the HLA-G1 and HLA-G5 isoforms and evidence shows that all HLA-G isoforms are capable of modulating immune system response (62). On the other hand, 4H84 and MEM-G/1, the most used antibodies to recognize HLA-G isoforms, cross-react with class I HLA molecules (60). The antibodies developed to directly block the interaction between HLA-G and its receptors must be very specific: to block the interaction of HLA-G with ILT-2 and ILT-4, these antibodies must recognize the α3 domain and β2m, whereas to block the interaction of HLA-G with KIR2DL4, they must recognize the α2 domain.

Some studies have shown that HLA-G can increase PD-1 expression on T cells through its interaction with ILT-2, which may lead to upregulation of PD-1, CTLA-4, and TIM-3 in ILT-2-positive CD8+ T cells, but not in ILT-2-negative CD8+ or CD4+ T cells (63). The above confirms the immune response modulating effect of the interaction between HLA-G and its receptors. In contrast, T cell stimulation with HLA-G-positive extracellular vesicles (EVs) increased the expression of other immune checkpoint molecules only in ILT-2-negative CD8+ T cells, indicating that HLA-G may act on different T cell populations depending on whether it is administered by EVs or in free soluble form (63); therefore, blockade of the PD-1/PD-L1 interaction, together with the blockade of HLA-G/ILT-2 interaction, could rescue the antitumor functions of T cells. In addition, it is possible to speculate that inhibition of HLA-G-positive EV secretion could restore the cytotoxic activities of T cells against tumor cells. Two clinical studies are currently underway in cancer patients: the first for the treatment of patients with different types of cancer using the human mAb TTX-080 directed against HLA -G, and the second using the bispecific antibody JNJ-78306358 directed against HLA-G and CD3 (64).

### 2.4 Lymphocyte activation gene 3

While immunotherapies directed against CTLA-4 and PD-1/PD-L1 have shown remarkable success in treating certain cancers, they are not effective for all patients and many of them do not benefit from it. This lack of response has led to the search for other suppressor mechanisms and inhibitory receptors expressed in the tumor microenvironment. LAG-3 gene, known as CD223, like CTLA-4 and PD-1, inhibits the CD8+ T cells function and increases the suppressive activity of Treg lymphocytes (64).

The type I transmembrane protein LAG-3 has four immunoglobulin (Ig)-like domains. Its extracellular region shares approximately 20% homology with CD4 and binds HLA-II with higher affinity than CD4 (65). LAG-3 is expressed on activated CD8+ and CD4+ T cells, regulatory CD4+ T cells (Treg), activated natural Treg cells (nTreg), CD4+ FoxP3-induced Treg lymphocytes (iTreg), B lymphocytes, plasmacytoid DCs, and a subset of NK cells. LAG-3 blockade in Treg cells abrogate their suppressive function, and in murine models, LAG-3 deficient CD4+ and CD8+ T cells showed increased T cell expansion, suggesting that a role of LAG-3 in the negative regulation of T cell homeostasis by regulating the clonal expansion of activated T cells (66, 67).

**Figure 5:**
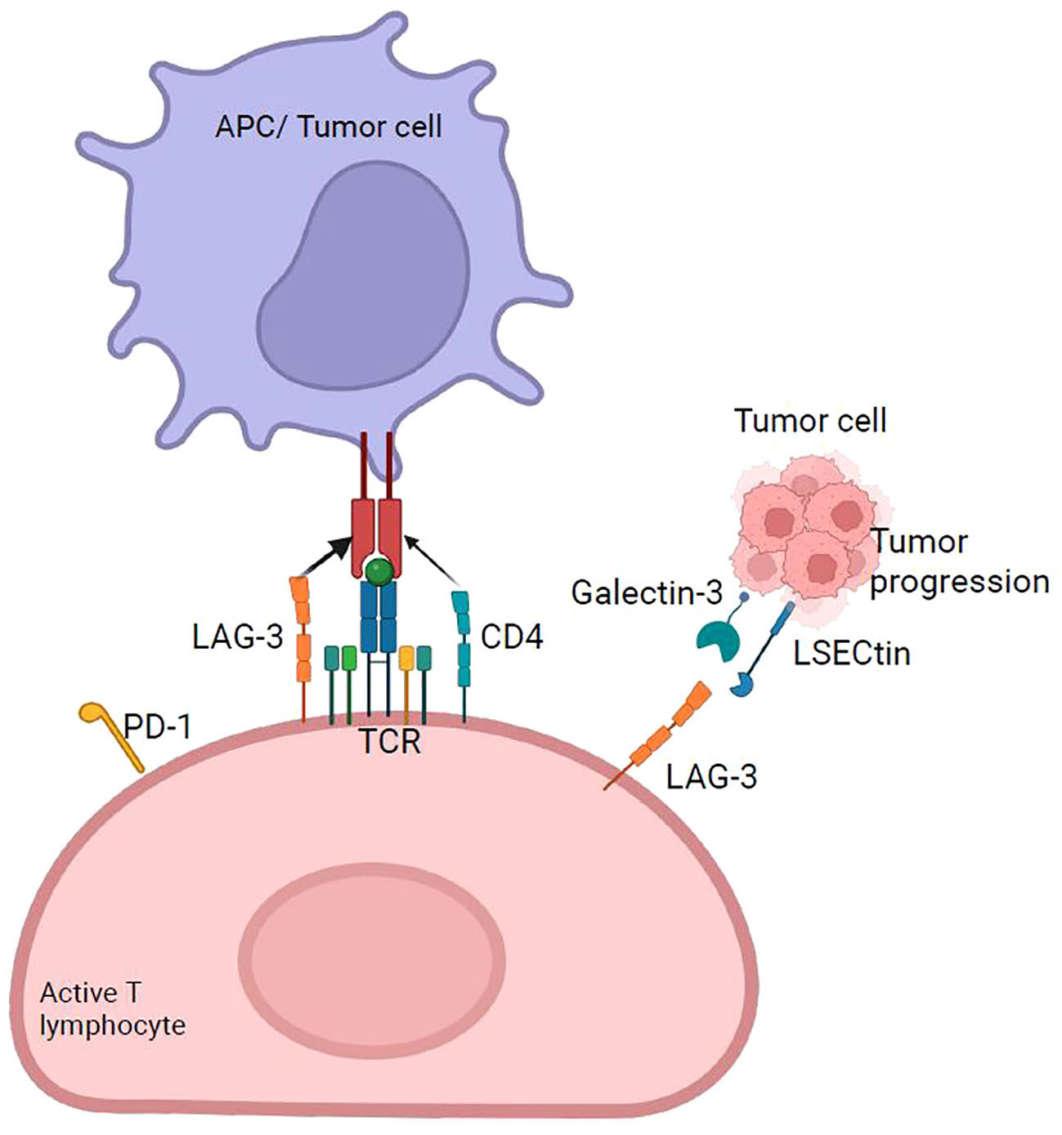
LAG-3 checkpoint. LAG-3 expressed primarily on activated T cells binds to HLA-II with higher affinity than CD4 and generates the overexpression of immunoregulatory cytokines, such as IL-10 and TGF-β, which suppress tumor-specific T cells. It acts synergistically with PD-1 to suppress antitumor immunity. The main ligand of LAG-3 is HLA-II, and four others have been discovered: FGL-1, galectin-3, LSECtin, and α-syn.

It has been established that LAG-3 binds to HLA-II with higher affinity than CD4. Nevertheless, the fact that LAG-3 also affects the function of CD8+ T cells and NK cells, which do not interact with HLA-II, suggests the existence of alternative ligands for LAG-3 (66) (**Figure 5**). Two additional LAG-3 ligands were described: LSECtin and galectin-3 (68). LSECtin, a member of the C-type lectin family, is expressed on the DC membrane, inhibits T cell responses (**Figure 5**), and its expression in melanomas is associated with tumor growth promotion, whereas blocking its expression slows tumor growth (68). Galectin-3 belongs to the galectin family of soluble lectins that bind to galactose. They are secreted by various tumors, and tumor stromal cells. Another functional ligand for LAG-3 is the fibrinogen-like protein 1 (FGL1), secreted by the liver and human tumor cells. The FGL1/LAG-3 interaction could potentially contribute to the resistance of anti-PD therapy in human cancers, and thus, FGL1 overexpression in the plasma of cancer patients is associated with poor prognosis and resistance to anti-PD-1/PD-L1 therapy. Moreover, blockade of the FGL1-LAG-3 interaction in the tumor microenvironment improve the antitumor immunity and enhances T cell responses as an immune escape mechanism and may have potential target for design a novel cancer immunotherapy (69).

LAG-3 also has a soluble form (sLAG-3) that provides greater immune control and regulation in the stroma or tumor microenvironment.it has been suggested that sLAG-3 alters the monocytes differentiation into macrophages or DCs, producing APCs with low immunostimulatory capacity (70). The soluble form of LAG-3 could be a prognostic biomarker in breast (71) and gastric cancer (72).

Exposure to the tumor microenvironment induces sustained expression of LAG-3, resulting in alterations in cell proliferation and cytokine production. LAG-3 expression levels and LAG-3+ cell infiltration in cancer is associated with tumor progression, poor prognosis, and unfavorable clinical outcomes in several human tumor types (73). LAG-3 signaling pathways and their interaction with other immune checkpoints have not yet been elucidated. Nevertheless, it is known that the intracellular region of LAG-3 contains a KIEELE motif essential for its function of inhibiting the proliferation of effector CD4+ T cells by preventing their entry into the S phase of the cell cycle, suppressing T cell expansion (74).

Although it is not clear whether PD-1 and LAG-3 share the same mechanisms of action, it has been reported synergy between LAG-3 and PD-1 to inhibit effector immune responses (75), as a high percentage of LAG-3+ and LAG-3− tumor-infiltrating CD8+ T cells expressing PD-1 has been observed (76). On the other hand, LAG-3 is co-expressed with PD-1 in CD8+ T cell (**Figure 5**), and synergy between LAG-3 and PD-1 has been demonstrated to enhance antitumor CD8+ T cell responses when LAG-3 and PD-1 pathways are simultaneously blocked (59, 77), suggesting that the role of LAG-3 as an immune checkpoint in regulating T cell function is more subtle than that of other immune checkpoint molecules.

LAG-3 has been proposed as a therapeutic target because its expression is overregulated in anergic T cells; it is suggested that using mAbs to block its expression could reverse this anergic state. Moreover, considering that PD-1 and LAG-3 are co-expressed on anergic T cells, the use of combined immunotherapy directed against PD-1/LAG-3 could reverse the anergic state in a chronic inflammatory environment (78).

#### 2.4.1 Inhibitory antibodies against LAG-3

Inhibitory effects of LAG-3 on effector T and Treg cells mediated by cross-linking of LAG-3 with the CD3/TCR complex, that inhibits TCR-induced T cell proliferation, cytokine production, and calcium entry (79). The exact molecular mechanism of cross-linking in LAG-3 signaling is unclear, but it depends on the LAG-3 intracellular KIEELE motif conserved in all species (67). Several LAG-3 inhibitors have been developed: relatlimab (BMS-986016), binds with high affinity to CD8+ T. and Treg cells. In preclinical studies, 48 melanoma patients were treated with relatlimab alone or in combination with nivolumab, showed overall response rates of 12.5%, with no adverse responses (80). Another LAG-3 antibody under study in combination with pembrolizumab is MK-4280. Preclinical studies demonstrate a synergistic antitumor activity by blocking LAG-3 binding to HLA-II and PD-1 to PD-L1 and PD-L2 with the use of LAG525 in combination with spartalizumab (anti-PD-1) (42). Etigilimab (OMP-313M32), was evaluated in 18 patients with advanced cancer and no limiting toxicities or adverse responses were observed (81). A clinical trial to analyze if the use of sym022, a mAb anti-LAG-3 antibody, in humans, if safe and tolerable, for patients with locally advanced/irresectable or metastatic lymphomas or malignant solid tumors refractory to available therapies, showing an undesirable outcome with high progression and adverse effect rates (NCT03489369).

### 2.5 T-cell immunoglobulin-3

T-cell immunoglobulin-3 (TIM-3), discovered in 2001 (82), is a type I transmembrane protein initially identified as a cell surface marker specific for IFN-γ production. TIM-3 expression is regulated by the interaction between the transcription factor T-bet, and the TIM-3 promoter (83). TIM-3 is expressed on activated T cells, in Treg lymphocytes, NK cells, monocytes, macrophages, DCs, and in various tissues (liver, small intestine, thymus, kidney, spleen, lung, muscle, and brain) (40). The principal ligand binding to TIM-3 is galectin-9, which interacts with the carbohydrate motif on TIM-3, triggering a calcium influx into Th1 cells and ultimately inducing apoptosis. In addition to galectin-9, various other ligands for TIM-3 have been identified, including phosphatidyl serine, high-mobility group box-1 (HMGB1), and carcinoembryonic antigen cell adhesion molecule 1 (CEACAM1) (82).

**Figure 6:**
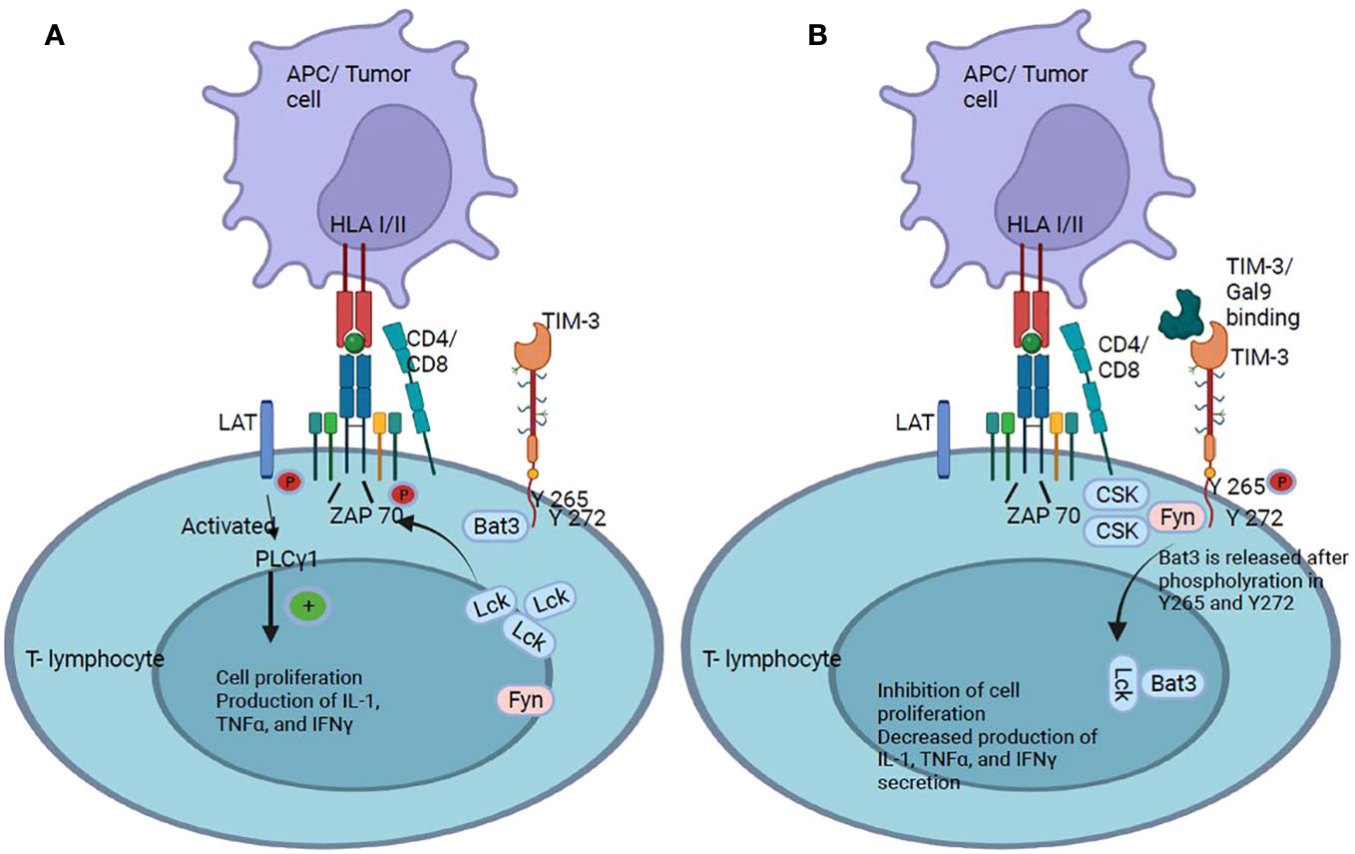
TIM-3 checkpoint. **(A)** TIM-3 has five tyrosine residues in its cytoplasmic tail, of which Y265 and Y272 are the most important for signal transduction. It has been shown that the cytoplasmic protein Bat3 is able to modulate cell proliferation: Bat3 binds to TIM-3 and protects T cells from signaling. **(B)** When TIM-3 binds to galectin-9, Bat3 dissociates from TIM-3, and signaling pathways are activated that lead to the inhibition of T cell proliferation and suppression of IL-2, TNFα, and IFNγ production.

Signaling through TIM-3 depends on the phosphorylation of tyrosine 265 by inducible T-lymphocyte kinase, which leads to the release of Bat3, which decreases T cell activation and, consequently, antitumor immunity (82). TIM-3 acts as a negative regulator of responses mediated by Th1 T cells and CTLs. When TIM-3 does not bind to any of its ligands, residues Y265 and Y272 of the cytoplasmic tail interact with Bat3, which facilitates the recruitment of the kinase Lck. Following the initial steps, the Lck groups phosphorylate the CD3 domain of the TCR. This phosphorylation event triggers the recruitment of the tyrosine kinase Zap 70, which in turn, phosphorylates the linker adaptor protein (LAT). Once activated, serves as a platform for recruiting and activating the signaling effector phospholipase Cγ1 (PLCγ1), which plays a pivotal role in the signaling cascade by generating second messengers like inositol 1,4,5-triphosphate (IP3) and diacylglycerol (DAG). These second messengers in turn activate downstream events. IP3 induces intracellular calcium release and DAG activates the protein kinase C family, activating multiple signaling pathways such as nuclear factor of activated T-cells (NFAT), mitogen-activated protein kinase/extracellular signal-regulated kinase (MEK/ERK), and nuclear factor kappa B (NF-kB). Activation of these pathways, resulting in T cell response, modulation of T cell proliferation, and production of immune signaling molecules such as IL-2, tumor necrosis factor (TNFα), IFNγ (**Figure 6A**) (83, 84). When TIM-3 binds to galectin-9, Y265 and Y272 in the cytoplasmic tail of TIM-3 are phosphorylated and Bat3 and Lck were released. Tyrosine kinase Fyn, recruited in the cytoplasmic tail of TIM-3, promote C-terminal tyrosine phosphorylation inhibiting its catalytic activity and further activating signaling pathways to inhibit T cell proliferation and inhibition of IL-2, TNFα, and IFNγ production (83) (**Figure 6B**).

In animal models, combined immunotherapy against PD-1 and TIM-3 to treat experimental and carcinogen-induced tumors suggests that these agents, in combination, could be very effective and well tolerated. Although no dramatic therapeutic effects were observed with the use of these antibodies, the impact of combined therapy (anti-TIM-3 and anti-PD-1) is significantly better than that of separate agents for cancer treatment in the carcinogen induction model (85).

#### 2.5.1 Inhibitory antibodies against TIM-3

T cell immunoglobulin and the mucin protein-3 (TIM-3) overexpression is associated with T cell dysfunction and correlates with PD-1 expression in CD8+ T cells, and in some depleted T cells lacking PD-1 expression (81, 86). In different cancer models, including melanoma, NSCLC, and follicular lymphoma, dual blockade of PD-1 and TIM-3 is much more effective in restoring the effector function of T cells (87–89). The potential of dual PD-1/TIM-3 blockade to increase tumor antigen-specific cell responses *in vitro* has been evidenced *in vivo* by reduced tumor growth (90). The antibodies MBG453, TSR-022, BMS-986258, and INCAGN02390 are some of the inhibitors that have been developed against TIM-3; among them, TSR-022, a humanized mAb IgG4k isotype that binds with high affinity to TIM-3, is the most advanced although the specificity of its ligand is not known exactly (81). TSR-022 is being studied as monotherapy or combined with other antibodies. This is the first human study to evaluate the action of TSR-022 antibody. The study will be developed in two parts. The first will consist of dose increase to determine the recommended dose of TSR-022 for the second part (RP2D), that will focus on dose expansion to evaluate the antitumor activity of TSR-022 combined with TSR-042 or docetaxel, and as monotherapy (NCT03680508) (clinicaltrials.gov)

### 2.6 Killer immunoglobulin-like receptors

Natural killer (NK) cells are innate immune cells with spontaneous cytotoxic activity against stressed cells such as tumor or virus-infected cells. Unlike T or B cells, they express numerous activating or inhibitory receptors, including KIR. These activating and inhibitory receptors are expressed in random combinations and give rise to functionally distinct NK cell populations. The lytic potential of NK cells and their ability to produce interferon-gamma (IFN-g) are regulated by receptor-ligand bindings, by type I interferons, and by cytokines such as IL-2, IL-15, and IL-18 secreted by other immune cells. NK cells can activate their effector functions spontaneously, and to prevent self-reactivity, they express inhibitory receptors that recognize self-HLA-I molecules. Although, NK cells belong to innate immunity, they can recognize their target cells by integrating signals generated by the interaction of their inhibitory and activating receptors with their respective ligands to trigger the secretion of cytokines and chemokines and the cytotoxic activity to eliminate virus-infected or tumor cells whose immune evasion mechanism consists of the downregulating HLA-I expression (91).

**Figure 7:**
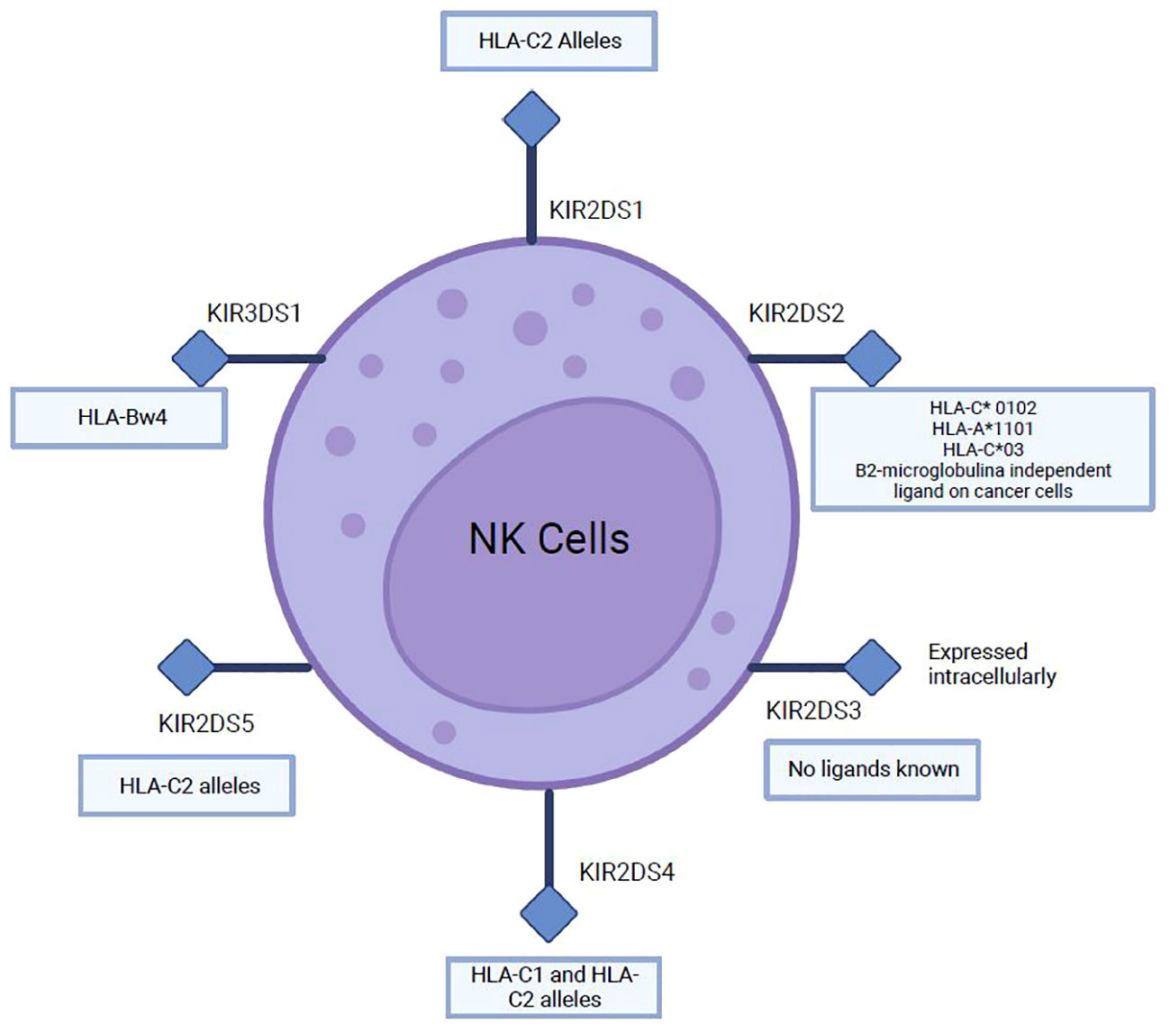
Main ligands of several activating KIRs (2DS and 3DS).

The KIR gene family consists of 15 genes and two pseudogenes classified into activating KIRs (KIR2DS and KIR3DS) (**Figure 7**) and inhibitory KIRs (KIR2DL and KIR3DL). These KIR receptors recognize specific motifs of some HLA-I epitopes with varying affinities and modulate NK cell activation and inhibition (92, 93); they are part of the NK cell receptor complex. Fourteen KIRs have been characterized in humans that contain two (2D) or three (3D) immunoglobulin-like extracellular domains and a cytoplasmic tail that can be long (L) or short (S). Six of these receptors are activating, while the rest are inhibitory KIRS. Activating KIRs have a truncated cytoplasmic tail, lack ITIM motifs, and have a positively charged amino acid (Lys or Arg) in their transmembrane domain, with which they associate with the DAP12 molecule, which contains in its structure immunoreceptor tyrosine-based activation motifs (ITAMs). Inhibitory KIRs are type 2 transmembrane receptors containing two immunoreceptor tyrosine-based inhibition motifs (ITIMs) to activate downstream signaling pathways, which inhibit NK cell function. Inhibitory KIR receptors bind to different HLA-I allotypes; however, the ligands for most activating KIR receptors are still unknown (92–95).

Ligands of several inhibitory and activating KIRs have been described, which recognize several HLA-I epitopes important for NK cell function (96). As for inhibitory KIRs, KIR2DL1 recognizes HLA-C2 while KIR2DL2 and KIR2DL3 recognize C1 and some C2 HLA-C allotypes; KIR3DL1 binds to the Bw4 epitope, which constitutes about 40% of HLA-B allotypes and some HLA-A molecules (A*23, A*24, A*25, and A*32). HLA-B epitopes are the most important ligands of KIR3DL1, recently it has been suggested that HLA-A and HLA-B allotypes may be equally important for NK cell function (96). Ligands for activating KIR are described in (**Figure 7**). Many different strategies have been identified to increase NK cell activity against cancer; however, relatively few have specifically targeted the activating KIRs (97). The cross-linking of activating KIRs may enhance NK cell activity, but the lack of mAbs selective for activating KIRs has hindered the development of this strategy (95, 97).

#### 2.6.1 Inhibitory antibodies against KIRs

KIR receptors play an important role as receptors for HLA-I molecules. Because HLA-I and KIR molecules are encoded in different chromosomes, a wide variety of related KIR/HLA-I genotypes are generated, so they are studied together in association with different pathologies, such as transplants, reproductive disorders, and cancer (98). Several inhibitory antibodies against KIR receptors have been produced, including lirilumab, which had two clinical trials (clinicaltrials.gov): one to evaluate its safety and tolerability when administered in combination with ipilimumab in subjects with advanced solid tumors (NCT01750580), and the other to evaluate its safety, tolerability, and antitumor activity when administered in combination with nivolumab (NCT01714739). Similarly, the humanized anti-KIR mAbs IPH2101 was used in a phase II multicenter study in patients with latent multiple myeloma to determine its antitumor activity (NCT00999830).

### 2.7 CD137 (4-1BB)

**Figure 8:**
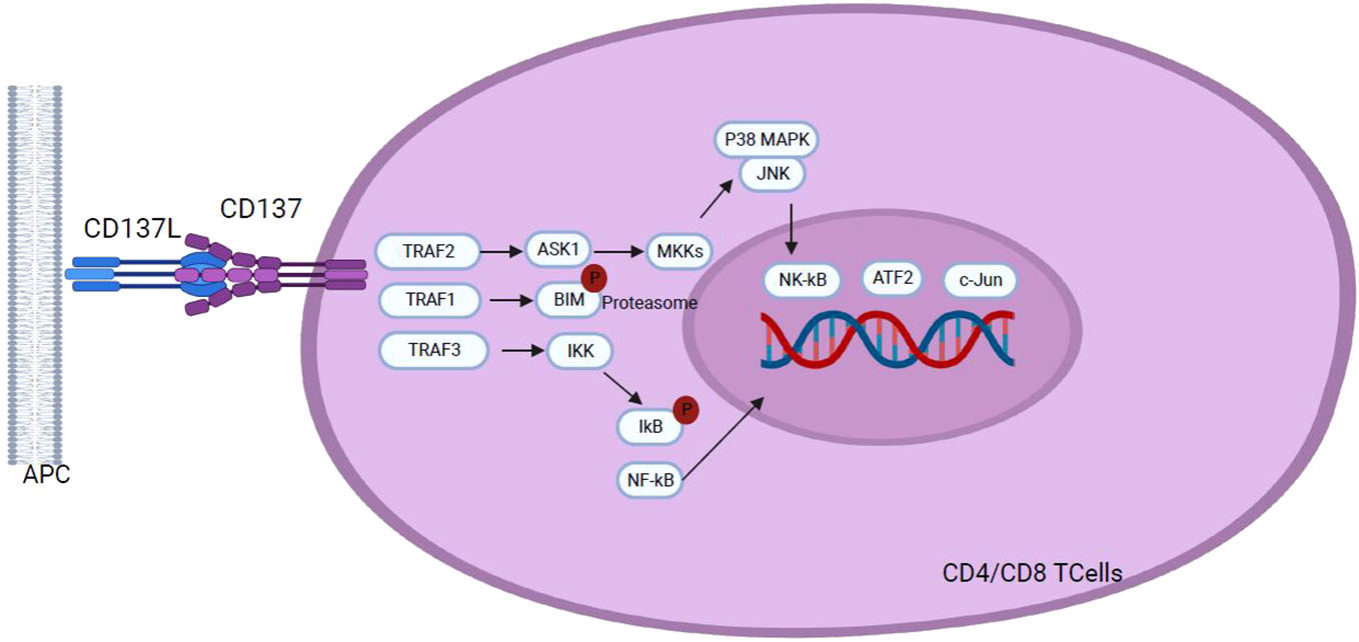
Following interaction with CD137L, CD137 signaling initiates through the recruitment of TRAF1 and TRAF2, and TRAF3 in a hypothetical scenario. TRAF proteins assemble into homo- or hetero-trimers which recruit cIAP1/2. This interaction plays a pivotal role in activating downstream effector signals orchestrating a cascade of events that transmit signals across various pathways to the nucleus, including NF-κB, ERK, p38 MAPK, and JNK pathways. Activation of these pathways results in the upregulation of anti-apoptotic proteins, T cell proliferation, differentiation, effector functions and overall survival, and down regulation of the pro-apoptotic protein Bim, highlighting the role of CD137 signaling in the modulation of key cellular processes with implications for the immune response homeostasis.

The CD137 receptor (also known as 4-1BB), initially discovered on activated T cells, belongs to the superfamily of TNF receptors. It is an inducible co-stimulatory receptor expressed on immune cells such as stimulated and activated T-CD4+ and T-CD8+ cells, as well as on activated NK cells, neutrophils, and mature DCs (17, 99). This type II transmembrane glycoprotein binds to its ligand, 4-1BBL or CD137L, expressed on the surface of professional APCs, such as activated macrophages, DCs, and B cells (100, 101). Unlike the other immune checkpoint molecules previously discussed, 4-1BB is an activating immune checkpoint on T cells. TCR stimulation followed by CD3 signaling induces transient expression of CD137, which, upon binding to its ligand, triggers a signaling cascade in T cells, resulting in cytokine secretion, anti-apoptotic molecules expression, and increased cell effector function, which favors Th1 responses. Activated T and NK cells exhibit CD137 transient expression while resting T cells do not express it. As CD137 expression is induced by TCR stimulation and CD3 signaling (102), activation of these signaling pathways results in increased proliferation, differentiation, and effector functions of T-CD4+ and T-CD8+ cells and induce the expression of anti-apoptotic proteins (**Figure 8**) (100). The cellular response to CD137/CD137L binding varies depending on the CD137-positive cell, as its expression is not limited to T cells but also observed on NK cells, monocytes, DCs, endothelial cells, and cancer cells (100).

#### 2.7.1 Inhibitory antibodies against CD137 (4-1BB)

It has been shown that the impact of CD137 therapy is greater when combined with other immune checkpoint inhibitor therapies (103). Anti-CD137 therapy combined with anti-PD-1 reduces tumor incidence more effectively than when administered separately, demonstrating the potential agonism of this marker (104, 105). A new bispecific antibody (4-1BB×PD-L1) called ABL503 was designed exclusively to activate CD137 signaling only in the context of PD-L1, thereby also blocking PD-1/PD-L1 signaling. This bispecific antibody was developed to avoid liver toxicity generated by anti-CD137 mAb and was found to exert strong antitumor therapeutic efficacy with a low risk of liver toxicity (106).

## 3 Current challenges of immune checkpoint inhibitor therapy

### 3.1 Immune-related adverse events

Despite advances made in integrating diverse antibody therapies to target immune checkpoints in different cancer types, a significant number of patients experience unaltered tumor progression (107), and although immune checkpoint inhibitors (ICI) therapies are increasing, a pivotal challenge in clinical application is the emergence of IRAEs (108). The use of antibodies for ICI leads to a distinct spectrum of toxicities compared to conventional treatments, which can result in heightened immune activity against normal organs such as the skin, colon, liver, lungs, kidneys, and heart, contributing negatively to patient outcomes (109). IRAEs incidence varies according to the antibody used (anti-CTLA-4 or anti-PD-1/PD-L1) and the treatment scheme (monotherapy or combined therapy). Generally, patients treated with anti-CTLA-4 antibodies exhibit a higher incidence of IRAEs compared to those treated with anti-PD-1/PD-L1 antibodies. Surprisingly, only three drugs -Ipilimumab, nivolumab, and pembrolizumab- account for almost 60% of reported cases of ICI-related adverse events (110). In addition, many studies indicate that some IRAEs may positively influence the efficacy of ICI. For instance, melanoma patients developing vitiligo or endocrine complications demonstrate enhanced tumor response and survival. Similarly, patients experiencing thyroiditis after PD-1 or PD-L1 blockade exhibit prolonged overall survival compared to their IRAE-negative counterparts (110).

### 3.2 Resistance to immune check point inhibitors

While immune checkpoint inhibitors have revolutionized the landscape of cancer treatments, addressing refractory disease remains a pivotal challenge (111). Despite the success of these therapies, a significant proportion of patients fail to achieve a sustained, long-term response. The efficacy of ICI therapies, particularly with PD-1 monotherapies demonstrates considerable benefits, with response rates ranging from ~40% to 70% in specific cancer types such as melanoma, Merkel cell carcinoma, Hodgkin’s lymphoma, and tumors with high microsatellite instability (MSI) (112). However, response rates success varies to a more modest range of 10-25% in other cancer types (113).

The success of Immune checkpoint inhibitor therapy is particularly noteworthy in melanoma, with approved treatments now encompassing anti-PD-1 agents such as nivolumab and pembrolizumab, the anti-CTLA-4 inhibitor ipilimumab, and combination therapies featuring both anti-PD-1 and CTLA-4 inhibitors like nivolumab-ipilimumab. Remarkably, long-term survival data for melanoma patients treated with ipilimumab indicates that 20% of patients exhibit sustained disease response persisting over 5 to 10 years from the initiation of therapy (114). The pembrolizumab response rate for melanoma patients at 3 years was 33% and 70% to 80% of patients that exhibit an initial positive response sustain the clinical response over time (115).

However, while the overall survival rate reaches 49% at 6.5 years for advanced melanoma patients treated with combination therapies like nivolumab-ipilimumab, a considerable proportion of patients either derive minimal benefit from immunotherapy or experience early disease relapse/progression within the initial months of treatment, leading to significant reduced survival rates (111–113), underscoring the need of understanding response dynamics to identify factors affecting response rates among melanoma patients treated with immunotherapies.

The lack of response to ICI treatments is perceive as a form of resistance broadly categorized into primary resistance, observed in patients who do not respond and progress rapidly with ICIs; and acquired resistance observed in patients who initially respond but eventually experience clinical and/or radiological progression of the disease (113).

Numerous intrinsic (intracellular and intratumor) or extrinsic (systemic) events may contribute to the observed ICI resistance, encompassing molecular alterations, cellular dynamics, metabolic adaptations, and microbiome effects, impacting the T-cell activation process. Regulation of these immune checkpoints are pivotal to shape resistance mechanisms related to the T-cell activation process that are categorized into specific types of resistance, including those related to antigen recognition, T-cell migration and/or infiltration, and T-cell effector functions (116).

Combination therapies of ICI with chemotherapy in lung (117), breast and gastric cancer has been developed with the aim of delaying or preventing the emergence of resistance to ICI therapies. Simultaneously extensive investigations into predictive biomarkers for the initial response to ICI have been conducted, seeking to adapt treatments according to resistance mechanisms and thus improve response rates in patients.to tailor treatments to resistance mechanisms to improve response rates in patients (113, 118). For example, in the context of EGFR-mutated lung cancer, as a model for targeted molecular therapy, the identification of resistance to first-generation of tyrosine kinase inhibitors (TKIs) revealed a secondary mutation in EGFR that prompted the development of third-generation inhibitors, such as Osimertinib specifically designed to target EGFR-T790M (119). New studies searching for mechanisms of acquired resistance to Osimertinib have been conducted and generated additional therapeutic successes (113, 120).

Significant challenges in the identification of those patients who will respond or not respond to immunotherapy, primarily due to the diverse response patterns observed with ICI. This diversity may manifest spatially, showing varied responses in different lesions, and/or temporally, as seen in disease progression. Within a specific patient, this heterogeneity may manifest as mixed responses, oligometastatic progression, and/or stability with isolated progression (112). On the other hand, it is crucial to underscore the limited information available on acquired therapy resistance, as it is not consistently reported, and distinct characteristics between different tumor types, difficult the prediction of treatment responses. Further studies with long term follow-ups specifically focusing on acquired resistance rates among responders are needed, to better quantify acquired resistance (113).

## 4 Discussion

Interactions between tumor cells and their microenvironment play a crucial role in cancer progression. In this microenvironment, tumor cells employ many strategies to evade immune surveillance, including altered expression of immunomodulatory molecules on immune cells, especially T and NK cells, as well as macrophages (121). These immune checkpoint molecules maintain immune homeostasis, but are also associated with suppression of immune responses, preventing immune destruction of tumor cells. In this context, immunotherapy based on ICI has radically transformed the therapeutic approach to cancer (122). In this review, we mainly focus on checkpoints that are widely distributed in various immune cells, exposing their regulatory functions in the context of cancer, and exploring their clinical application along with the main antibodies investigated for immunotherapy.

Despite advances in research and confirmation that checkpoint blockade is effective for cancer treatment; several difficulties have been identified in the clinical application of this strategy. First, some tumors, due to their low immunogenicity, do not respond effectively to checkpoint blockade, leading to resistance to therapy. This resistance, whether primary or acquired, may be due to compensatory mechanisms, such as positive regulation of alternative immune checkpoints like BTLA, A2AR, B7-H4, NOX2, HO-1 and SIGLEC7 (122), which although not mentioned in this review, are also considered promising targets for immune therapy, in addition to PD-1, CTLA-4 and TIM-3. Secondly, elucidation of molecular mechanisms of action remains a challenge due to heterogeneity in studies, ranging from the sensitivity of different antibodies to the techniques used and the types of tissues investigated. This poses challenges in the accurate identification of biomarkers to detect patients who will respond to ICI and in the management of adverse effects of these treatments. Currently, immune-related adverse events (IRAEs) are a potential clinical biomarker of response to ICI in patients. However, the cause of IRAEs is still not fully understood, which represents a challenge in clinical treatment with ICI, as these side effects can significantly affect the therapeutic effect and prognosis of patients. It has been shown that the combined use of ICI can lead to a higher incidence of IRAEs than single ICI therapy, which also varies according to the type of malignancy and the ICI used (123), as certain immune checkpoints can be used in synergy with PD-1 and TIM-3 as discussed above. In addition, the use of some immune checkpoint antibodies can affect the immune function of other normal tissues at the same time such as ipilimumab, which is widely used and has been associated with severe IRAEs, which can be attributed to broad-spectrum immune suppression (124). As we have seen in this review, the wide expression of immune checkpoints in various immune cells (**Table 1**) leads us to consider that it is important to consider the immune response elicited by ICI throughout the immune system to avoid the breakdown of immune balance that leads to IRAEs. With future research and systematic literature reviews addressing the different mechanisms and applications of immune checkpoints, we will be able to explore the diversity of ICI, identify new therapeutic targets, guide combination therapy with ICIs and predict therapeutic response more accurately.

## 5 Conclusions

Immunotherapy based on immune checkpoints blockade has revolutionized the field of cancer treatment, not only in early stages but also in resistant cases. We review the origins of these immune checkpoints and the development of some of the most prominent ICIs. Despite a relatively slow clinical adoption, this review underscores the promising potential of this approach, supported by numerous studies demonstrating improved responses through single or combination therapies.

Further preclinical and clinical studies are needed to gain a more complete understanding of how the immune system initiates and maintains an effective antitumor response, the systemic effects of other immunotherapies, and the adverse effects that may be generated. Ultimately, these efforts will allow understanding the systemic response that defines effective therapy for patients to help guide and inform future therapies. One key conclusion is the need for further exploration through preclinical and clinical studies to comprehensively understand the initiation and sustenance of an efficient antitumor immune response. This includes investigating the systemic effects of various immunotherapies and the potential adverse reactions they may elicit. Overall, this review highlights the transformative role and impact of immune checkpoint-based immunotherapy in cancer treatment, the importance of continued research to optimize its effectiveness while minimizing adverse effects and the development of key Immune Checkpoint Inhibitors (ICIs).

## Author contributions

LM-G: Conceptualization, Investigation, Writing – original draft. PM-C: Investigation, Validation, Writing – review & editing. JR-G: Conceptualization, Funding acquisition, Project administration, Supervision, Writing – review & editing. AT-S: Investigation, Writing – review & editing.
