# Supplementary material for: The role of immune checkpoints in antitumor response: a potential antitumor immunotherapy
Source: Front Immunol. 2023 Dec 15;14:1298571. doi: 10.3389/fimmu.2023.1298571 (PMC10757365; doi:10.3389/fimmu.2023.1298571)
Supplement: Supplementary file 1 [file Table_2.docx]

**Tabla 2. Clinical trials based on main FDA-approved immune immune checkpoint inhibitors (ICIs) in human cancers.**

| **NCT Number** | **Study Status** | **Conditions** | **Drug** | **Phase** | **Completion Date** |
| --- | --- | --- | --- | --- | --- |
| NCT03406715 | Terminated | Small Cell Lung Cancer\|  Lung Cancer\|  Relapsed Small Cell Lung Cancer | Nivolumab-Ipilimumab | Phase2 | 16/05/2022 |
| NCT03026166 | Terminated | Small Cell Lung Cancer | Ipilimumab\| Nivolumab\| Rovalpituzumab tesirine | Phase1\|Phase2 | 3/07/2019 |
| NCT03663166 | Terminated | Carcinoma, Non-Small-Cell Lung | ipilimumab\| Nivolumab | Phase1\|Phase2 | 22/10/2021 |
| NCT02498600 | Active NR | Fallopian Tube Carcinoma  Ovarian Carcinoma  Peritoneal Carcinoma | Ipilimumab\| Nivolumab | Phase2 | 31/12/2023 |
| NCT03215706 | Active NR | Non-Small Cell Lung Cancer | Ipilimumab\| Nivolumab | Phase3 | 19/01/2026 |
| NCT02231749 | Active NR | Renal Cell Carcinoma | Nivolumab\| Ipilimumab | Phase3 | 7/08/2027 |
| NCT02872116 | Active NR | Gastric Cancer  Gastroesophageal Junction Cancer  Esophageal Adenocarcinoma | Nivolumab\|Ipilimumab | Phase3 | 31/05/2024 |
| NCT04034927 | Active NR | Fallopian Tube Endometrioid cancer Fallopian Tube Serous Adenocarcinoma  Ovarian Endometrioid Tumor  Ovarian Serous Adenocarcinoma Endometrioid Adenocarcinoma | Tremelimumab | Phase2 | 21/09/2024 |
| NCT03071406 | Active NR | Merkel Cell Carcinoma\|  Skin Cancer | Nivolumab\|Ipilimumab | Phase2 | 2024-07 |
| NCT02785952 | Active NR | Squamous Cell Lung Carcinoma | Ipilimumab\| Nivolumab | Phase3 | 1/04/2024 |
| NCT02919683 | Active NR | Head and Neck Cancer | DRUG: Nivolumab\|DRUG: Ipilimumab | Phase2 | 2024-04 |
| NCT03430063 | Terminated | Advanced Non-Small Cell Lung Carcinoma | Ipilimumab | Phase2 | 27/10/2021 |
| NCT04088500 | Terminated | Renal Cell Carcinoma | Nivolumab\| Ipilimumab | Phase2 | 15/11/2021 |
| NCT02750514 | Terminated | Advanced Cancer | Nivolumab\|Relatlimab\| Ipilimumab\|DRUG: BMS-986205 | Phase2 | 29/01/2020 |
| NCT03168464 | Terminated | Non-Small Cell Lung Cancer Metastatic | Ipilimumab\| Nivolumab | Phase1\|Phase2 | 11/03/2022 |
| NCT03693846 | Terminated | Colon Adenocarcinoma  Rectum Adenocarcinoma | Nivolumab\| Ipilimumab | Phase2 | 6/03/2021 |
| NCT04434560 | Terminated | Brain Metastases, Adult | Nivolumab\| Ipilimumab | Phase2 | 17/06/2021 |
| NCT03515629 | Terminated | Non-small Cell Lung Cancer | Pembrolizumab | Phase3 | 29/07/2021 |
| NCT03141177 | Active NR | Renal Cell Carcinoma | Nivolumab\| Ipilimumab | Phase3 | 17/04/2024 |
| NCT03143153 | Active NR | Various Advanced Cancer | Nivolumab\|Ipilimumab | Phase3 | 13/01/2025 |
| NCT03347123 | Terminated | Solid Tumors | Nivolumab\| Ipilimumab\| Lirilumab | Phase1\|Phase2 | 29/01/2021 |
| NCT03043599 | Active NR | Small Cell Lung Cancer | Ipilimumab\| Nivolumab | Phase1\|Phase2 | 2023-12 |
| NCT02953457 | Active NR | Ovarian Serous Adenocarcinoma Fallopian Tube Carcinoma  Ovarian Carcinoma  Peritoneal Carcinoma | Durvalumab\| Tremelimumab | Phase2 | 15/12/2023 |
| NCT03203473 | Active NR | Renal Cancer | Ipilimumab\| Nivolumab | Phase2 | 30/11/2024 |
| NCT01769222 | Terminated | Various Lymphomas | Ipilimumab | Phase1\|Phase2 | 2015-06 |
| NCT02892734 | Terminated | Breast Carcinoma | Ipilimumab\| Nivolumab | Phase2 | 4/02/2019 |
| NCT03522584 | Terminated | Head and Neck Squamous Cell Carcinoma | Durvalumab\| Tremelimumab | Phase1\|Phase2 | 19/01/2022 |
| NCT03816332 | Active NR | Cutaneous Melanoma  Merkel Cell Carcinoma  Metastatic Basal Cell Carcinoma | Ipilimumab\|Nivolumab | Phase1 | 22/09/2024 |
| NCT01377389 | Terminated | Prostate Cancer | Ipilimumab | Phase2 | 7/04/2017 |
| NCT02576509 | Active NR | Hepatocellular Carcinoma | Nivolumab | Phase3 | 31/01/2024 |
| NCT02498600 | Active NR | Fallopian Tube Carcinoma  Ovarian Carcinoma  Peritoneal Carcinoma | Ipilimumab\| Nivolumab | Phase2 | 31/12/2023 |
| NCT03215706 | Active NR | Non-Small Cell Lung Cancer | Ipilimumab\| Nivolumab\| | Phase3 | 19/01/2026 |
| NCT03117049 | Active NR | Non-Small Cell Lung Cancer | Bevacizumab | Phase3 | 2024-05 |
| NCT02231749 | Active NR | Renal Cell Carcinoma | Nivolumab\| Ipilimumab\| | Phase3 | 7/08/2027 |
| NCT02872116 | Active NR | Gastric Cancer  Gastroesophageal Junction Cancer Esophageal Adenocarcinoma | Nivolumab\|Ipilimumab\| | Phase3 | 31/05/2024 |
| NCT03071406 | Active NR | Merkel Cell Carcinoma  Skin Cancer | Nivolumab\| Ipilimumab\| | Phase2 | 2024-07 |
| NCT02632409 | Active NR | Various Advanced Cancer | Nivolumab\| | Phase3 | 15/05/2027 |
| NCT02785952 | Active NR | Squamous Cell Lung Carcinoma | Ipilimumab\| Nivolumab\| | Phase3 | 1/04/2024 |
| NCT02919683 | Active NR | Head and Neck Cancer | Nivolumab\| Ipilimumab | Phase2 | 2024-04 |
| NCT03141177 | Active NR | Renal Cell Carcinoma | Nivolumab\|Ipilimumab | Phase3 | 17/04/2024 |
| NCT03404960 | Active NR | Pancreatic Adenocarcinoma | Niraparib + Nivolumab\| Niraparib + Ipilimumab | Phase1\|Phase2 | 2026-06 |
| NCT03341936 | Active NR | Squamous Cell Carcinoma of the Head and Neck | Nivolumab\| Lirilumab | Phase2 | 6/07/2026 |
| NCT03370276 | Active NR | Oropharynx Squamous Cell Carcinoma  Squamous Cell Carcinoma of the Larynx Cell Carcinoma. | Nivolumab\| Cetuximab | Phase1\|Phase2 | 2024-01 |
| NCT02664181 | Active NR | Lung Cancer\|Non-small Cell Lung Cancer | DRUG: Nivolumab\| | Phase2 | 14/02/2024 |
| NCT03043599 | Active NR | Small Cell Lung Cancer\|Extensive-stage Small Cell Lung Cancer | Ipilimumab\| Nivolumab | Phase1\|Phase2 | 2023-12 |
| NCT03496662 | Active NR | Pancreatic Ductal Adenocarcinoma | DRUG: Nivolumab | Phase1\|Phase2 | 14/10/2024 |
| NCT03203473 | Active NR | Renal Cancer | Ipilimumab\| Nivolumab | Phase2 | 30/11/2024 |
| NCT03502330 | Active NR | Advanced Melanoma  Non-small Cell Lung Cancer  Renal Cell Carcinoma | Cabiralizumab\|Nivolumab | Phase1 | 2027-10 |
| NCT02257528 | Active NR | Cervical Adenocarcinoma\| | Nivolumab | Phase2 |  |
| NCT02998528 | Active NR | Non Small Cell Lung Cancer | Nivolumab\|Ipilimumab | Phase3 | 8/11/2028 |
| NCT03816332 | Active NR | Cutaneous Melanoma  Merkel Cell Carcinoma | Ipilimumab\| Nivolumab | Phase1 | 22/09/2024 |
| NCT03382561 | Active NR | Lung Small Cell Carcinoma | Nivolumab | Phase2 | 31/12/2023 |
| NCT03713593 | Active NR | Carcinoma, Hepatocellular | pembrolizumab | Phase3 | 29/08/2024 |
| NCT02766582 | Active NR | Ovarian Cancer | Pembrolizumab | Phase2 | 2023-12 |
| NCT02609503 | Active NR | Head and Neck Cancer | Pembrolizumab | Phase2 | 30/11/2023 |
| NCT02808143 | Active NR | Bladder Carcinoma  Urothelial Carcinoma | Pembrolizumab | Phase1 | 2023-02 |
| NCT02586207 | Active NR | Head and Neck Cancer  Squamous Cell Carcinoma  Oral Cavity Cancer  Oropharynx Cancer  Larynx Cancer  Laryngeal Cancer | pembrolizumab | Phase1 | 2024-09 |
| NCT03004183 | Active NR | Non-small Cell Lung Cancer  Triple-negative Breast Cancer | Pembrolizumab | Phase2 | 2023-11 |
| NCT02673333 | Active NR | Adrenocortical Carcinoma | Pembrolizumab | Phase2 | 2024-02 |
| NCT03675737 | Active NR | Stomach Neoplasms | Pembrolizumab | Phase3 | 28/09/2024 |
| NCT03149822 | Active NR | Metastatic Renal Cell Carcinoma | Pembrolizumab | Phase1\|Phase2 | 31/12/2023 |
| NCT02949219 | Active NR | Metastatic Small Intestinal Adenocarcinoma | Pembrolizumab | Phase2 | 31/03/2024 |
| NCT02546986 | Active NR | Carcinoma, Non-Small-Cell Lung | Pembrolizumab | Phase2 | 3/04/2024 |
| NCT03602586 | Active NR | Ovarian Carcinoma | Pembrolizumab | Phase2 | 22/09/2024 |
| NCT02818920 | Active NR | Non-small Cell Lung Carcinoma | Pembrolizumab | Phase2 | 2026-03 |
| NCT03631706 | Active NR | Non-small Cell Lung Cancer | Pembrolizumab | Phase3 | 24/03/2024 |
| NCT02641093 | Active NR | Head and Neck Cancer | Pembrolizumab | Phase2 | 2/11/2025 |
| NCT03396471 | Active NR | Carcinoma, Unspecified Site | Pembrolizumab | Phase2 | 1/01/2024 |
| NCT02957968 | Active NR | Breast Adenocarcinoma  Negative Breast Cancer | Pembrolizumab | Phase2 | 28/02/2025 |
| NCT03260894 | Active NR | Renal Cell Carcinoma (RCC) | Pembrolizumab | Phase3 | 31/12/2024 |
| NCT02830594 | Active NR | Gastric Adenocarcinoma  Gastric Squamous Cell Carcinoma  Gastroesophageal Junction Adenocarcinoma | Pembrolizumab | Phase2 | 30/12/2023 |
| NCT02971761 | Active NR | Triple-Negative Breast Carcinoma | Pembrolizumab | Phase2 | 28/12/2023 |
| NCT02811861 | Active NR | Renal Cell Carcinoma | Pembrolizumab | Phase3 | 31/07/2024 |
| NCT03062358 | Active NR | Carcinoma, Hepatocellular | pembrolizumab | Phase3 | 31/10/2024 |
| NCT02316002 | Active NR | Oligometastatic Non-small Cell Lung Cancer (NSCLC) | Pembrolizumab | Phase2 | 30/09/2023 |
| NCT03971474 | Active NR | Lung Non-Small Cell Carcinoma | Pembrolizumab\| Ramucirumab | Phase2 | 1/11/2023 |
| NCT04676412 | Active NR | Non-small Cell Lung Cancer | Pembrolizumab | Phase3 | 30/11/2023 |
| NCT02853331 | Active NR | Renal Cell Carcinoma | Pembrolizumab | Phase3 | 29/12/2023 |
| NCT03358472 | Active NR | Head and Neck Cancer | Pembrolizumab | Phase3 | 27/12/2023 |
| NCT04696055 | Active NR | Hepatocellular Carcinoma | DRUG: Pembrolizumab | Phase2 | 15/05/2024 |
| NCT02648477 | Active NR | Triple-Negative Breast Carcinoma | Pembrolizumab | Phase2 | 30/12/2023 |
| NCT03829332 | Active NR | Non-small Cell Lung Cancer | Pembrolizumab | Phase3 | 8/03/2024 |
| NCT02964078 | Active NR | Kidney Cancer | Pembrolizumab | Phase2 | 2024-04 |
| NCT03898180 | Active NR | Urothelial Carcinoma | Pembrolizumab | Phase3 | 1/07/2024 |
| NCT03783078 | Active NR | Merkel Cell Carcinoma | Pembrolizumab (MK-3475) | Phase3 | 16/06/2032 |
| NCT04581824 | Active NR | Lung Cancer, Non-Small Cell | Dostarlimab\| Pembrolizumab | Phase2 | 20/10/2025 |
| NCT03631784 | Active NR | Non-small Cell Lung Cancer | Pembrolizumab | Phase2 | 12/03/2024 |
| NCT02296684 | Active NR | Cancer of Head and Neck | Pembrolizumab (MK-3475) | Phase2 | 31/12/2025 |
| NCT02730546 | Active NR | Gastric Cardias Adenocarcinoma  Gastric Cancer | Pembrolizumab | Phase1\|Phase2 | 15/05/2024 |
| NCT04027946 | Active NR | Lung Cancer\|Non-Small Cell Lung Cancer\|Adenocarcinoma of Lung | pembrolizumab | Phase2 | 31/03/2024 |
| NCT02621398 | Active NR | Non-Small Cell Lung Cancer | Pembrolizumab | Phase1 | 30/09/2023 |
| NCT03142334 | Active NR | Renal Cell Carcinoma | Pembrolizumab | Phase3 | 28/12/2025 |
| NCT03040999 | Active NR | Head and Neck Neoplasms | Pembrolizumab | Phase3 | 29/06/2024 |
| NCT03684785 | Terminated | Metastatic Merkel Cell Carcinoma Squamous Cell Carcinoma\|  Melanoma  Head and Neck Squamous Cell Carcinoma  Solid Tumors | Pembrolizumab\|Cemiplimab | Phase1\|Phase2 | 30/03/2022 |
| NCT04305795 | Active NR | Head and Neck Squamous Cell Carcinoma\|Metastatic Head-and-neck Squamous-cell Carcinoma\|Locally Advanced Cutaneous Squamous Cell Carcinoma\|Metastatic Cutaneous Squamous Cell Carcinoma | Pembrolizumab\| Cemiplimab | Phase1\|Phase2 | 2025-06 |
| NCT03563716 | Active NR | Non-small Cell Lung Cancer | Atezolizumab\|Tiragolumab | Phase2 | 31/03/2025 |
| NCT03853707 | Active NR | Anatomic Stage IV Breast Cancer AJCC v8\|Metastatic Triple-Negative Breast Carcinoma\|Prognostic Stage IV Breast Cancer AJCC v8 | Atezolizumab | Phase1\|Phase2 | 21/12/2023 |
| NCT04081688 | Active NR | Refractory Lung Non-Small Cell Carcinoma\|Stage IV Lung Cancer AJCC v8 | Atezolizumab | Phase1 | 30/04/2024 |
| NCT02873195 | Active NR | Metastatic Colorectal Carcinoma\|Recurrent Colorectal Carcinoma\|Refractory Colorectal Carcinoma\|Stage IV Colorectal Cancer AJCC v7\|Stage IVA Colorectal Cancer AJCC v7\|Stage IVB Colorectal Cancer AJCC v7 | Atezolizumab\|Bevacizumab | Phase2 | 1/07/2024 |
| NCT03735121 | Active NR | Non-Small Cell Lung Cancer | Atezolizumab | Phase3 | 31/12/2024 |
| NCT03689855 | Active NR | Non-small Cell Lung Cancer\|Non Small Cell Lung Cancer\|NSCLC | Ramucirumab\|Atezolizumab | Phase2 | 25/04/2024 |
| NCT02724878 | Active NR | Advanced Non-Clear Cell Kidney Cancer | Bevacizumab\|Atezolizumab | Phase2 | 2023-10 |
| NCT03201458 | Active NR | Gallbladder Carcinoma\|Metastatic Cholangiocarcinoma\|Stage III Intrahepatic Cholangiocarcinoma AJCC v8\|Stage IV Intrahepatic Cholangiocarcinoma AJCC v8\|Unresectable Cholangiocarcinoma | Atezolizumab | Phase2 | 13/02/2024 |
| NCT02927301 | Active NR | Non-Small Cell Lung Cancer | Atezolizumab (MPDL3280A) | Phase2 | 3/05/2024 |
| NCT04373369 | Active NR | Extensive-stage Small Cell Lung Cancer | Atezolizumab | Phase2 | 5/07/2025 |
| NCT03738228 | Active NR | Cervical Adenocarcinoma\|Cervical Adenosquamous Carcinoma\|Cervical Squamous Cell Carcinoma\|Stage IB2 Cervical Cancer AJCC v8\|Stage II Cervical Cancer AJCC v8\|Stage IIA Cervical Cancer AJCC v8\|Stage IIA1 Cervical Cancer AJCC v8\|Stage IIA2 Cervical Cancer AJCC v8\|Stage IIB Cervical Cancer AJCC v8\|Stage IIIB Cervical Cancer AJCC v8\|Stage IVA Cervical Cancer AJCC v8 | Atezolizumab | Phase1 | 21/09/2024 |
| NCT02118337 | Completed | Select Advanced Malignancies\|Kidney Cancer\|Clear Cell Renal Cell Carcinoma | Durvalumab\|Nivolumab | Phase1\|Phase2 | 17/03/2020 |
| NCT02701400 | Completed | Recurrent Small Cell Lung Carcinoma | Durvalumab\| Tremelimumab | Phase2 | 7/08/2020 |
| NCT03204812 | Completed | Castration-Resistant Prostate Carcinoma\|Metastatic Malignant Neoplasm in the Bone\|Prostate Adenocarcinoma\|Stage IV Prostate Cancer AJCC v8\|Stage IVA Prostate Cancer AJCC v8\|Stage IVB Prostate Cancer AJCC v8 | Durvalumab\| Tremelimumab | Phase2 | 13/04/2021 |
| NCT03373760 | Completed | Recurrent Squamous Cell Lung Carcinoma\|Stage IV Squamous Cell Lung Carcinoma AJCC v7 | BIOLOGICAL: Durvalumab\|OTHER: Laboratory Biomarker Analysis\|BIOLOGICAL: Tremelimumab | Phase2 | 29/03/2022 |
| NCT02953457 | Active NR | BRCA1 Gene Mutation\|BRCA2 Gene Mutation\|Ovarian Serous Adenocarcinoma\|Recurrent Fallopian Tube Carcinoma\|Recurrent Ovarian Carcinoma\|Recurrent Primary Peritoneal Carcinoma | Durvalumab\| Tremelimumab | Phase2 | 15/12/2023 |
| NCT02536794 | Completed | Estrogen Receptor Negative\|Estrogen Receptor Positive\|HER2/Neu Negative\|Recurrent Breast Carcinoma\|Stage IV Breast Cancer | Anti-B7H1 Monoclonal Antibody MEDI4736\| Tremelimumab | Phase2 | 22/01/2021 |
| NCT03522584 | Terminated | Metastatic Head and Neck Squamous Cell Carcinoma\|Recurrent Head and Neck Squamous Cell Carcinoma | Durvalumab\| Tremelimumab | Phase1\|Phase2 | 19/01/2022 |
